# Enhancement of diatom growth and phytoplankton productivity with reduced O_2_ availability is moderated by rising CO_2_

**DOI:** 10.1038/s42003-022-03006-7

**Published:** 2022-01-14

**Authors:** Jia-Zhen Sun, Tifeng Wang, Ruiping Huang, Xiangqi Yi, Di Zhang, John Beardall, David A. Hutchins, Xin Liu, Xuyang Wang, Zichao Deng, Gang Li, Guang Gao, Kunshan Gao

**Affiliations:** 1 State Key Laboratory of Marine Environmental Science & College of Ocean and Earth Sciences, Xiamen University, 361005 Xiamen, China; 2 School of Biological Sciences, Monash University, Clayton, VIC 3800 Australia; 3 Marine and Environmental Biology Section, Department of Biological Sciences, University of Southern California, Los Angeles, CA 90089 USA; 4 Key Laboratory of Tropical Marine Bio-resources and Ecology, South China Sea Institute of Oceanology, Chinese Academy of Sciences, 510301 Guangzhou, China; 5 Co-Innovation Center of Jiangsu Marine Bio-industry Technology, Jiangsu Ocean University, 222005 Lianyungang, China

**Keywords:** Climate-change ecology, Photosynthesis

## Abstract

Many marine organisms are exposed to decreasing O_2_ levels due to warming-induced expansion of hypoxic zones and ocean deoxygenation (DeO_2_). Nevertheless, effects of DeO_2_ on phytoplankton have been neglected due to technical bottlenecks on examining O_2_ effects on O_2_-producing organisms. Here we show that lowered O_2_ levels increased primary productivity of a coastal phytoplankton assemblage, and enhanced photosynthesis and growth in the coastal diatom *Thalassiosira weissflogii*. Mechanistically, reduced O_2_ suppressed mitochondrial respiration and photorespiration of *T. weissflogii*, but increased the efficiency of their CO_2_ concentrating mechanisms (CCMs), effective quantum yield and improved light use efficiency, which was apparent under both ambient and elevated CO_2_ concentrations leading to ocean acidification (OA). While the elevated CO_2_ treatment partially counteracted the effect of low O_2_ in terms of CCMs activity, reduced levels of O_2_ still strongly enhanced phytoplankton primary productivity. This implies that decreased availability of O_2_ with progressive DeO_2_ could boost re-oxygenation by diatom-dominated phytoplankton communities, especially in hypoxic areas, with potentially profound consequences for marine ecosystem services in coastal and pelagic oceans.

## Abstract (web summary)

Sun et al. investigate the effects of current ambient and potential future oxygen levels on phytoplankton growth and photosynthesis with field observations and mesocosm and lab experiments. Their results demonstrate positive effects of low O_2_ on phytoplankton growth, photosynthesis, and inorganic carbon acquisition at current and future high levels of CO_2_.

## Introduction

Hypoxic waters (defined as having dissolved O_2_ < 63 μM or 2 mg L^−1^) occur naturally in both open ocean and nearshore waters, and global warming, as well as anthropogenic eutrophication, have been increasing in their spatial extent and severity^1–4^. While hypoxia has often been considered exclusive to deeper waters, near-surface hypoxic waters (< 20 m) are often observed in estuaries^5^, coastal waters^6^, and upwelling regions^7^. Deoxygenation (DeO_2_) in these areas is predicted to accelerate with progressive ocean global changes, mainly due to ocean-warming^8^. Decreases in the dissolved O_2_ content of coastal seawaters are principally due to the heterotrophic degradation of dissolved organic matter associated with coastal eutrophication, resulting in low O_2_, low pH, and high CO_2_ conditions^9–11^. While such changes are measured in bulk seawater, their levels are not the same as those in the diffusion boundary layer (DBL) at the photosynthetic cell surface, but nonetheless modeling and direct measurement suggest that changes in the DBL exhibit the same trends, maintaining higher CO_2_ (lower pH) under elevated CO_2_ conditions or lower O_2_ under reduced O_2_ conditions^12,13^. Therefore, reduced O_2_ availability and increased CO_2_ (lowered pH) in seawater are co-varying drivers in the context of DeO_2_ and ocean acidification (OA)^14^. This combination has the potential to disturb the balance between photosynthetic energy supply and respiratory energy consumption in marine ecosystems, and can thus disrupt ecological services^3,15^.

Photosynthesis of phytoplankton is a major biogeochemical process that oxidizes the oceans, especially by the diatoms that have been estimated to contribute up to 52% of marine O_2_ production^16^ and that dominate the phytoplankton communities in hypoxic regions^17^. Photosynthesis of some diatoms appears to decrease with increased ratios of O_2_ to CO_2_ availability^18^, because carboxylation and oxygenation are catalyzed simultaneously by the central enzyme of photosynthesis ribulose-1,5-bisphosphate carboxylase/oxygenase (Rubisco), and these two reactions compete with each other at the active site of the enzyme to fix CO_2_ and to consume O_2_, respectively^19^. In common with most other phytoplankton, diatoms use energy-costly CO_2_ concentrating mechanisms (CCMs)^20^ to increase intracellular CO_2_ around the active site of Rubisco, minimizing competition from O_2_ and favoring efficient carboxylation^19^. It has been shown that increased seawater *p*CO_2_ at the levels projected for the end of this century can decrease CCM activity in diatoms and other microalgae^21,22^ and repress expression of CCM-related genes^23,24^. The energy savings and resources freed up from downregulation of the CCMs under elevated CO_2_ conditions could potentially increase primary production under low light levels^20,21,25,26^. However, under high light levels, excess photochemical energy has been suggested to act with acidic stress to enhance photoinhibition and therefore decrease primary productivity in surface phytoplankton communities^26^.

It is usually accepted that higher levels of chlorophyll *a* (Chl *a*) abundance are positively correlated with high primary productivity^27^. However, primary productivity per volume of water does not reflect photosynthetic activity or light use efficiency per Chl *a*, since higher O_2_ and low *p*CO_2_ are often found in waters of high Chl *a* concentrations^28^, and are supposed to reduce carboxylation or photosynthetic efficiency as aforementioned. These previous theoretical inferences^18^ along with our own fieldwork shown here and other observations showing higher levels of phytoplankton photosynthetic efficiency or biomass density in low O_2_ waters^29^ led us to hypothesize that a decreased *p*O_2_:*p*CO_2_ ratio in estuarine and coastal waters could enhance marine productivity and, that this effect of deoxygenation is due to differentially influenced physiological performances of CCMs and photorespiration, which together could act to increase diatom growth rates. OA entails both increased CO_2_ availability and acidic stress, and so may either decrease or increase photosynthetic efficiency and growth in diatoms, depending on taxonomic differences and environmental conditions^18,26,30–32^. In contrast, the interactions of increased CO_2_ and reduced O_2_ on phytoplankton have rarely been considered^18^. We present here a test of our hypothesis using a series of mesocosm and laboratory experiments that determined the combined effects of elevated CO_2_ and decreased O_2_ availability on diatom growth and photosynthesis.

## Results

### Field investigation

**Fig. 1:**
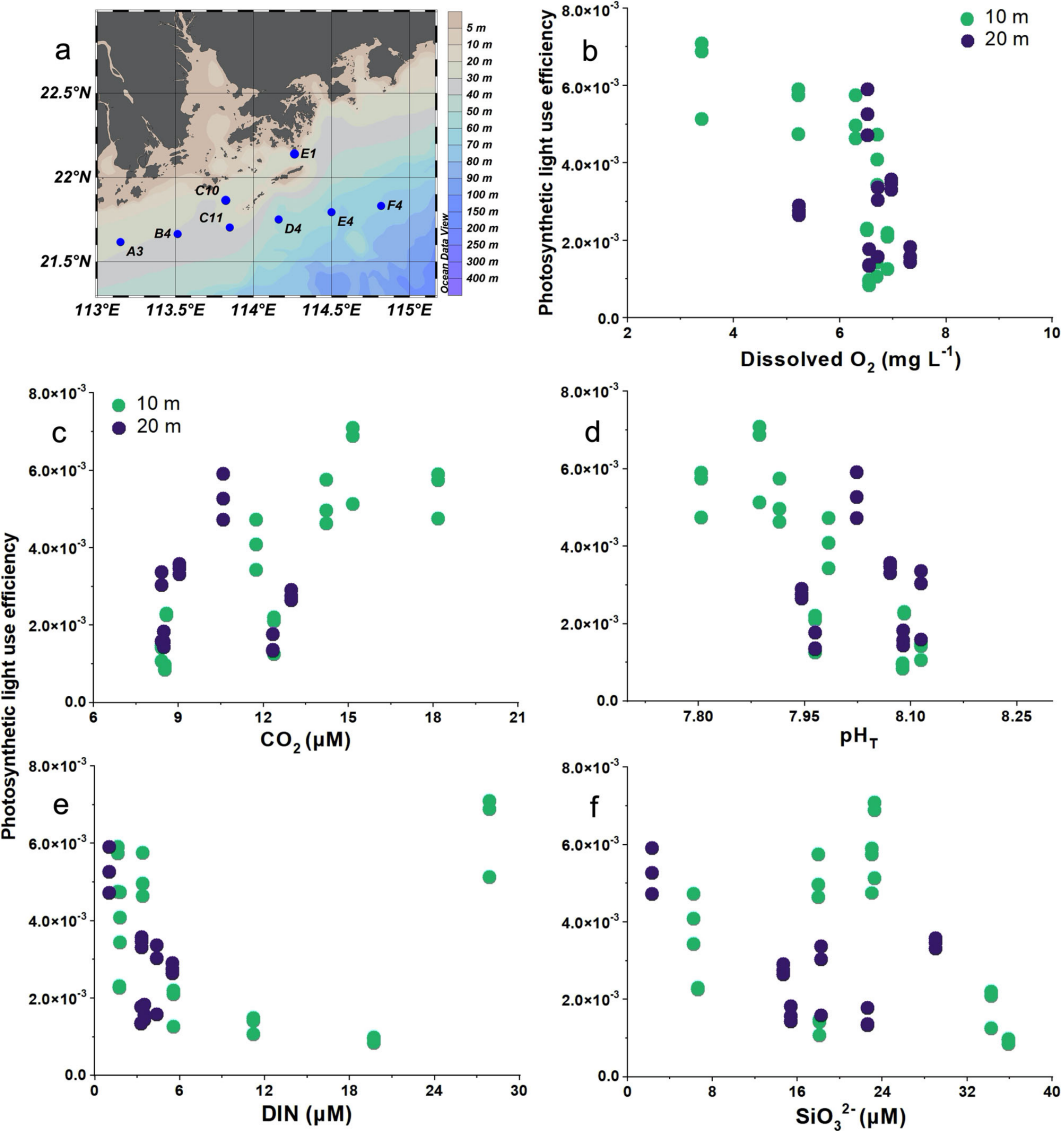
Field investigation of the Pearl River Estuary in the northern South China Sea. Photosynthetic light use efficiency [μmol C (μg Chl *a*)^−1^ h^−1^ (μmol photons m^−2^ s^−1^)^−1^] of phytoplankton assemblages in stations of Pearl River Estuary (**a**) as a function of dissolved O_2_ levels (mg L^−1^) (**b**), CO_2_ levels (μM) (**c**), pH_T_ (**d**), DIN (NO_3_^−^ + NO_2_^−^ + NH_4_^+^, μM) (**e**) and SiO_3_^2−^ (μM) (**f**). Samples were collected from 10 m (Green) and 20 m (Purple) depths in stations (Blue) of the Pearl River Estuary in the northern South China Sea (June 2015), detailed parameters for the field observations at the stations are shown in the Supplementary Table 1. Significant (*P* < 0.0001) negative (**b**, **d**) and positive (**c**) correlations and non-significant (**e**, **f**, *P* = 0.6671, 0.0707, Pearson correlation analysis, two-tailed) relationships with O_2_ (**b**), CO_2_ (**c**), pH_T_ (**d**), DIN (**e**) and SiO_3_^2−^ (**f**) are shown in Supplementary Table 2.

The aim of our field study was to examine whether photosynthetic activity correlates with levels of dissolved O_2_ (DO), a factor that has seldom been considered in the context of potential effects on oceanic primary productivity. Accordingly, environmental parameters that may influence photosynthetic carbon fixation were investigated at eight different stations in the Pearl River estuary (Fig. 1a, details in Supplementary Table 1). Photosynthetic light use efficiency [PLUE, μmol C (μg Chl *a*)^-1^ h^-1^ (μmol photons m^-2^ s^-1^)^-1^)] was derived from photosynthetic carbon fixation rates measured at low levels (photosynthesis-limiting, <100 and <60 μmol photons m^−2^ s^−1^ at 10 and 20 m, respectively) of incident sunlight. PLUE was significantly correlated with DO, CO_2_ and pH (Fig. 1b–d, Supplementary Table 2, *P* < 0.0001, *r* = −0.6120, 0.6589, −0.6463, respectively), but was unrelated to concentrations of dissolved inorganic nitrogen (DIN, NO_3_^−^ + NO_2_^−^ + NH_4_^+^) and SiO_3_^2−^ (Fig. 1e, f and Supplementary Table 2, *P* = 0.6671, 0.0707, respectively, Pearson Correlation Analysis). There was an obvious significant increase in PLUE with decreased DO. However, this negative correlation might be attributed to the positive effects of increased CO_2_ availability and other environmental factors (Fig. 1 and Supplementary Table 1). Therefore, we employed Partial Correlation Analysis to further exclude disturbance from other environmental factors on the correlation between DO and PLUE (see “Data Analysis” for details). These results again indicate a significant correlation of higher PLUE with lower DO (Supplementary Table 2, *P* = 0.0035, *r* = −0.4736), suggesting that DO could be one of the key drivers altering in situ photosynthesis and primary production.

### Natural phytoplankton assemblage mesocosm experiments

To test the responses of a natural coastal phytoplankton assemblage to different *p*O_2_:*p*CO_2_ combinations, we conducted a 30-liter mesocosm experiment under natural levels of sunlight and temperature (Supplementary Fig. 1a) with filtered (180 μm) seawater. While DO, CO_2_ levels, and pH varied over time, DO and CO_2_ remained significantly different between the low and high treatments (Supplementary Fig. 1b–d, *P* < 0.0001). Macronutrients in the mesocosms were consumed rapidly and became depleted within 5 days (Supplementary Fig. 2), with faster removal of the nutrients under low O_2_ conditions. This was especially obvious for NO_x_ and SiO_3_^2-^ (Supplementary Fig. 2a, c). In contrast, concentrations of chlorophyll *a* (Chl *a*) in the mesocosms increased rapidly and peaked within 3 days then declined, with higher concentrations of Chl *a* under low O_2_/high CO_2_ treatments at day 3 (Supplementary Fig. 2d, *P* = 0.0414, 0.1547 for LOAC and LOHC, respectively).

**Fig. 2:**
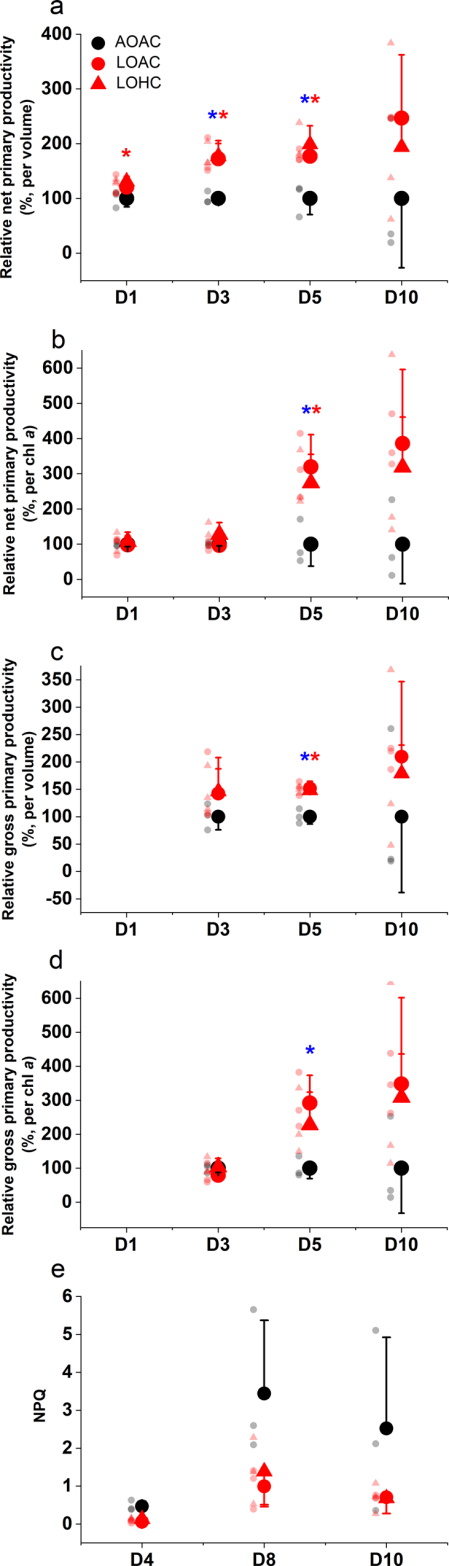
Photosynthetic carbon fixation and nonphotochemical quenching (NPQ) of natural phytoplankton assemblages grown under different O_2_ and CO_2_ treatments. (**a**) Net photosynthesis per volume of seawater (μmol C L^−1^ h^−1^), and (**b**) per Chl *a* (μmol C (μg Chl *a*)^−1^ h^−1^) measured at day 1, 3, 5, 10, as well as (**c**) gross photosynthesis per volume of seawater (μmol C L^−1^ h^−1^), and (**d**) per Chl *a* (μmol C (μg Chl *a*)^−1^ h^−1^) measured at day 3, 5, 10. In **a**–**d**, values are presented as % of rates under ambient CO_2_ and O_2_ levels, and the absolute values for the rates are shown in Supplementary Table 3. (**e**) Nonphotochemical quenching (NPQ) measured during the noon period at day 4, 8, 10. Black symbols represent ambient O_2_ (AO, ~213 μM) and red symbols low O_2_ (LO, ~57 μM); Circles represents ambient CO_2_ (AC, ~13 μM); triangles represent high CO_2_ (HC, ~27 μM). Mesocosms were incubated under incident sunlight and natural levels of temperature (Supplementary Fig. 1), and all data were obtained under growth conditions. Detailed information for the mesocosms experimental features are given in Supplementary Figs. 1 and 2. The values are the means with error bars indicating standard deviations of independent biological replicates (*n* = 3 mesocosms). Light-colored symbols are individual data corresponding to the treatments. Blue * and red * indicate significant differences (*P* < 0.05, LSD test) due to low O_2_ under ambient (LOAC) and elevated CO_2_ levels (LOHC), respectively, compared to the control treatment (AOAC).

During the mesocosm experiment, the net and gross photosynthetic rates were higher in the low O_2_ (LO)-grown than in the ambient O_2_ (AO)-grown phytoplankton assemblage under both ambient (AC) and high (HC) CO_2_ levels (relative changes are presented in Fig. 2a–d, absolute values in Supplementary Table 3 and specific *p* values in Supplementary Table 4), and these enhancements increased with time when NOx stocks diverged between the LO and AO treatments in the mesocosms (Fig. 2a–d and Supplementary Fig. 2a). Under AC, reduced O_2_ availability significantly enhanced the net photosynthetic rate per volume of seawater (Fig. 2a) at day 3 and day 5 (*P* = 0.0102, 0.0124), and such significant enhancement was also observed under high CO_2_ at day 1, 3, 5 (*P* = 0.0416, 0.0076, 0.0040). Similar trends were also found in Chl *a*-normalized net photosynthesis (Fig. 2b) under both LOAC and LOHC treatments, though significant enhancement was only observed at day 5 (*P* = 0.0145, 0.0359) and marginally significant enhancement at day 10 (*P* = 0.0529 for LOAC). Likewise, gross photosynthetic rates regardless of normalization units and CO_2_ levels were higher under reduced O_2_ levels (Fig. 2c, d). Elevated CO_2_ and the associated pH drop appeared to run counter to the stimulating effects of reduced O_2_, with lower mean values of photosynthetic rate in the LOHC treatment compared with the LOAC treatment under both normalized units (per water volume or per Chl *a*), but this was not statistically significant (Fig. 2a–d, *P* = 0.1270–0.9180, detailed *P* values in Supplementary Table 4).

**Fig. 3:**
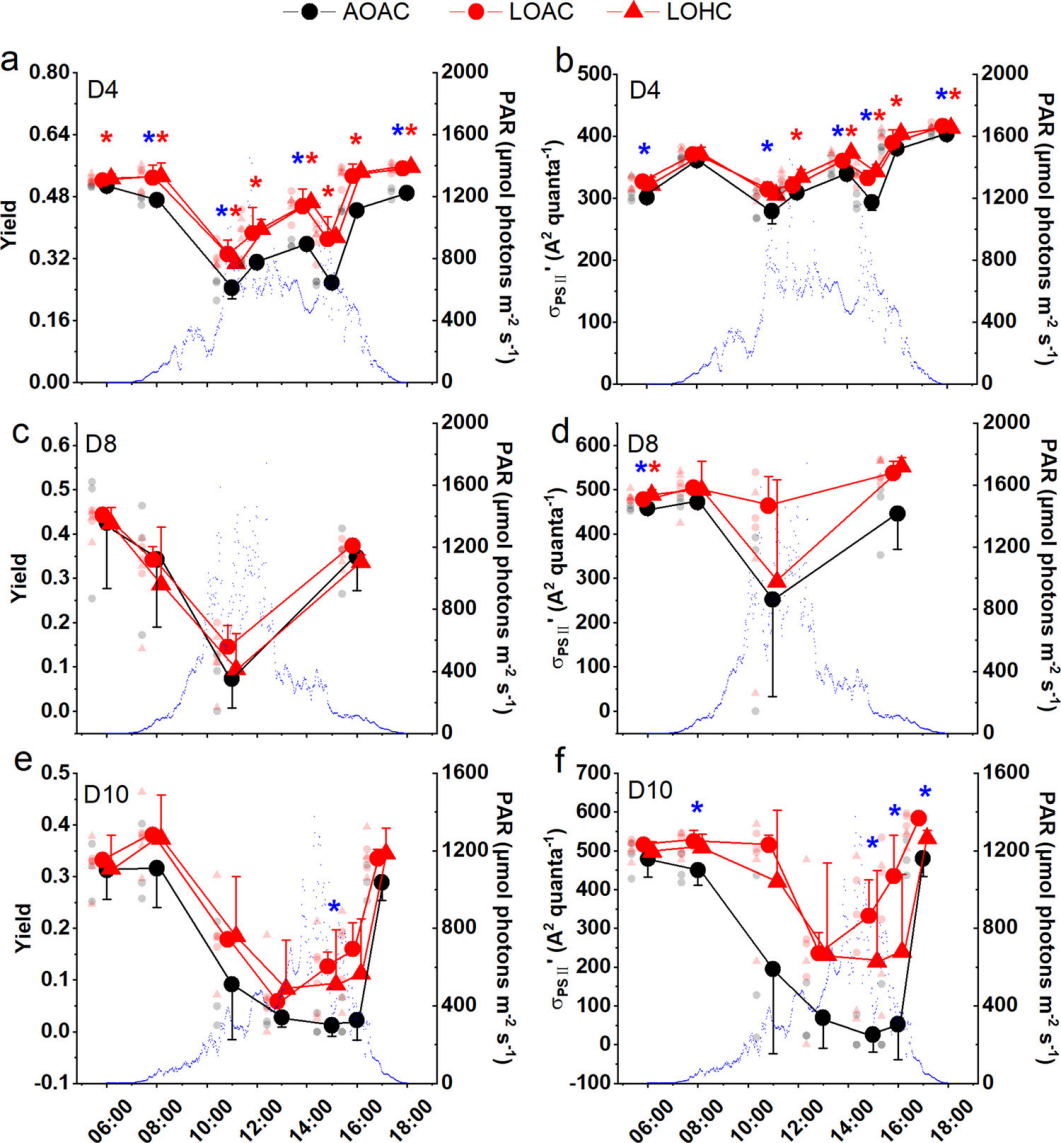
Diurnal changes in photosystem II (PSII) quantum yield (Yield) and the effective functional absorption cross-section of PSII (σ_PSII_´, A^2^ quanta^-1^) of phytoplankton assemblages grown under different O_2_ and CO_2_ treatments. Effective PSII quantum yield (**a**, **c**, **e**) and the effective functional absorption cross-section of PSII (**b**, **d**, **f**) at days 4, 8, and 10, respectively. Blue dots represent diel changes in photosynthetically active radiation (PAR, μmol photons m^−2^ s^−1^) during the experiment. Black symbols represent ambient O_2_ (AO, ~213 μM) and red symbols low O_2_ (LO, ~57 μM); circles represent ambient CO_2_ (AC, ~13 μM); triangles represent high CO_2_ (HC, ~27 μM). Detailed information for the mesocosms experimental features are given in Supplementary Figs. 1 and 2. The values are the means and the error bars represent standard deviations of independent biological replicates (*n* = 3 mesocosms). Light-colored symbols are individual data corresponding to the treatments. Blue * and red * indicate significant differences (*P* < 0.05, LSD test or Games–Howell test) caused by low O_2_ under ambient (LOAC) and elevated CO_2_ levels (LOHC), respectively, compared to the control treatment (AOAC).

Reduced O_2_ availability decreased nonphotochemical quenching (NPQ), an indicator of photosynthetic energy loss as heat dissipation and a signal of light stress (Fig. 2e), though only marginally significant changes were observed at day 4 (*P* = 0.0531 for LOAC and *P* = 0.086 for LOHC) and day 8 (*P* = 0.0552, 0.0932). In parallel, reduced O_2_ level increased photochemical yield (Yield, reflecting all processes downstream of PSII) and effective functional absorption cross-section (σ_PSII_´, an indicator of the efficiency of light capture) during the mesocosm experiment (Fig. 3, detailed *P* values in Supplementary Table 5). At day 4, reduced O_2_ increased the Yield significantly (Fig. 3a, *P* = 0.0002–0.0483) or marginally significantly (*P* = 0.0664–0.0813) under both CO_2_ levels, except at 15:00 (*P* = 0.1377 for LOAC). Meanwhile, reduced O_2_ significantly (Fig. 3b, *P* = 0.0004–0.0398) or marginally significantly (*P* = 0.0557–0.0780) enhanced σ_PSII_´ regardless of CO_2_ levels except at 08:00 (*P* = 0.3236 for LOAC and *P* = 0.3847 for LOHC), 12:00 (*P* = 0.2962 for LOAC) and 16:00 (*P* = 0.7898 for LOAC). Similar trends in these measurements were observed both on days 8 and 10 (Fig. 3c–f). These results suggested an enhanced energy transfer in LO-grown phytoplankton.

**Fig. 4:**
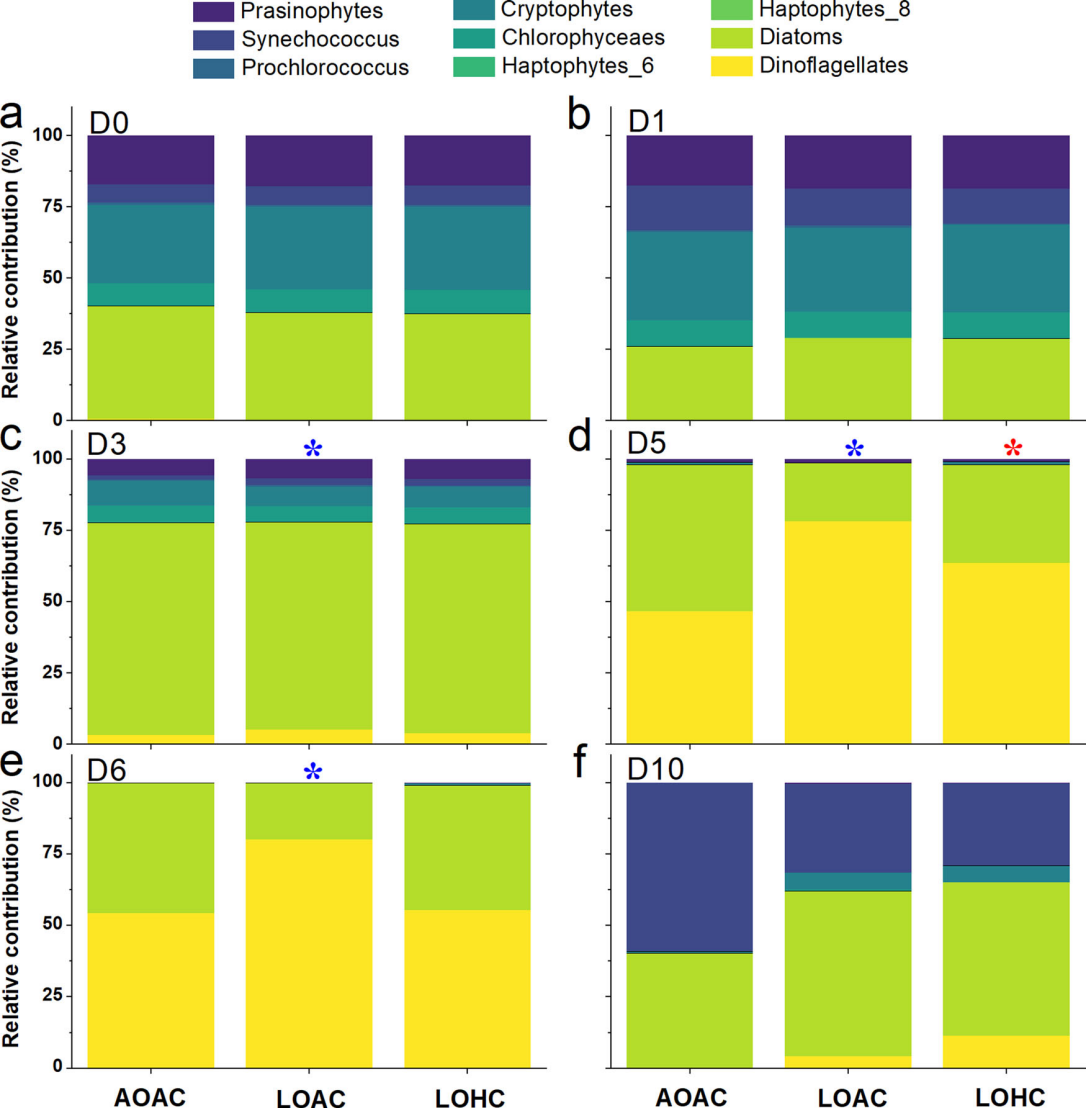
Taxonomic composition of phytoplankton assemblages in the mesocosms under three different O_2_/CO_2_ combinations. Proportions of different major phytoplankton groups are indicated in different colors on (**a**) day 0, (**b**) day 1, (**c**) day 3, (**d**) day 5, (**e**) day 6, and (**f**) day 10 under ambient (AO, ~213 μM) and low O_2_ levels (LO, ~57 μM) with ambient (AC, ~13 μM) and elevated CO_2_ levels (HC, ~27 μM). D0 represents the initial time of the experiment (22:00, December 27, 2018). Values represent the means of independent biological replicates (*n* = 3 mesocosms). Detailed information for the mesocosms experimental features are given in Supplementary Figs. 1 and 2. Blue * and red * indicate significant differences in proportions of dinoflagellates (*P* < 0.05, LSD test) caused by low O_2_ under ambient (LOAC) and elevated CO_2_ levels (LOHC), respectively, compared to the control treatment (AOAC).

Based on the CHEMTAX analysis, the phytoplankton community composition changed with time under the different O_2_ and CO_2_ combination treatments (Fig. 4). The diverse phytoplankton community was originally dominated by diatoms, cryptophytes, and prasinophytes, but then shifted to have higher proportions of dinoflagellates and the pico-cyanobacterium *Synechococcus* (Fig. 4) when nutrients were depleted (Supplementary Fig. 2). While diatoms continued as one of the dominant groups throughout the incubation period, the proportion of dinoflagellates obviously increased in the LOAC treatment (Fig. 4c–e, *P* = 0.0240, 0.0029, 0.0035, correspondingly). A similar trend was found in the LOHC treatment, although a significant increase was only observed at day 5 (Fig. 4d, *P* = 0.0405).

### Diatom culture experiment

Based on the field investigation and mesocosm experiment where diatoms were dominant, a diatom culture experiment was conducted to investigate photosynthetic performance, growth rate, and CCM efficiencies in the globally distributed coastal diatom *Thalassiosira weissflogii*. The cells were grown under four *p*O_2_:*p*CO_2_ combinations for over nine generations in laboratory culture. DO, carbonate chemistry and cell numbers were maintained in a stable range (with ~1000–5000 cells mL^−1^, Supplementary Fig. 3) by diluting the medium every 24 h without using aeration. Levels of DO, pH_T_, and CO_2_ in low O_2_ (LO) and high CO_2_ (HC) culture conditions differed from those in the ambient O_2_ (AO) and ambient CO_2_ (AC) treatments (Supplementary Fig. 3e, f and Supplementary Table 6).

**Fig. 5:**
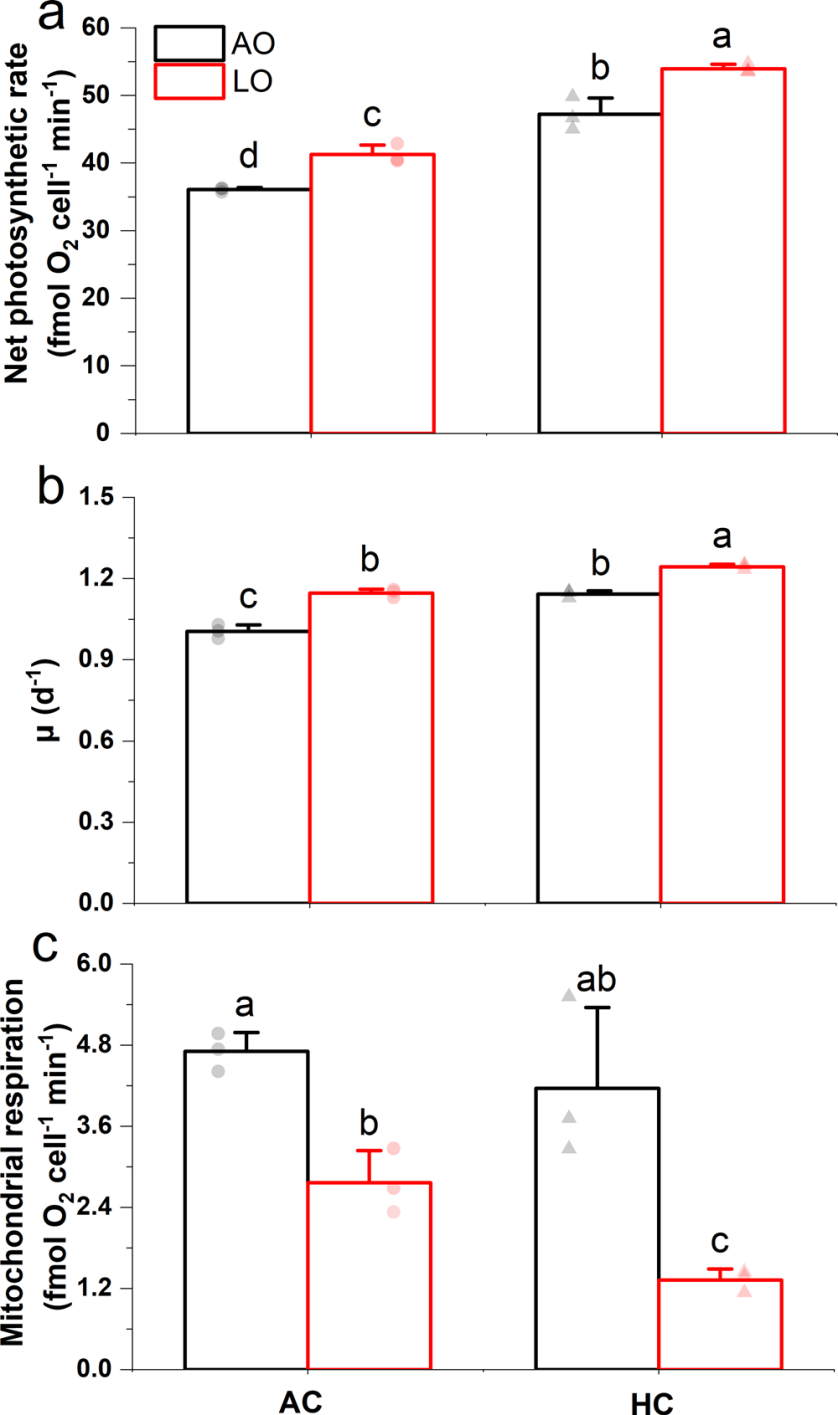
Photosynthetic O_2_ evolution, growth rates, and mitochondrial respiration rates of the diatom *Thalassiosira weissflogii* grown and measured under four different *p*O_2_/*p*CO_2_ combinations. (**a**) Net photosynthetic rates, (**b**) specific growth rates (μ), and (**c**) mitochondrial respiration rates of the cells grown and measured under ambient (AO, ~255 μM) and low O_2_ levels (LO, ~57 μM) with ambient (AC, ~15 μM) and elevated CO_2_ levels (HC, ~35 μM). The values are the means and the error bars represent standard deviations of independent biological replicates (*n* = 3 independent cultures). Light-colored symbols are individual data corresponding to the treatments. Different letters above the bars represent significant differences (*P* < 0.05, LSD test) among treatments. Detailed information for the experimental features and timing points for the above determinations are shown in Supplementary Fig. 3.

Reduced O_2_ levels significantly promoted net photosynthesis of the diatom by ~14% under both AC and HC levels (Fig. 5a, *P* = 0.0024, 0.0005). The absolute rates were higher by ~31% in the AOHC and by ~50% in the LOHC compared with the AOAC treatment (Fig. 5a, *P* < 0.0001, 0.0001), respectively. Decreased O_2_ concentration also increased the growth rate by ~14% under AC and only by 9% under HC (Fig. 5b, *P* < 0.0001, 0.0001). This suggests that there was substantially less enhancement of growth by reduced O_2_ under the influence of elevated CO_2_ with lowered pH.

Decreased O_2_ levels reduced mitochondrial respiration under the AC and HC levels by 41% and 68%, respectively (Fig. 5c, *P* = 0.0054, *P* < 0.0001), suggesting that mitochondrial respiration was suppressed by reduced O_2_ availability to a much greater extent under HC conditions. At the same time, LOAC- and LOHC-grown cells exhibited unchanged high values of photochemical efficiency compared with the cells grown under the AOAC treatment (Supplementary Fig. 4a, *P* = 0.4717, 0.9663). This indicates that the cells were maintaining a healthy physiological state with high light use efficiency. NPQ decreased significantly in low O_2_ treatments by 20% (*P* = 0.0016) and 32% (*P* < 0.0001) under AC and HC levels, respectively (Supplementary Fig. 4b), suggesting a more efficient energy transfer in LO-grown cells, which is consistent with the results from the mesocosm experiment using natural phytoplankton assemblages (Fig. 2e).

**Fig. 6:**
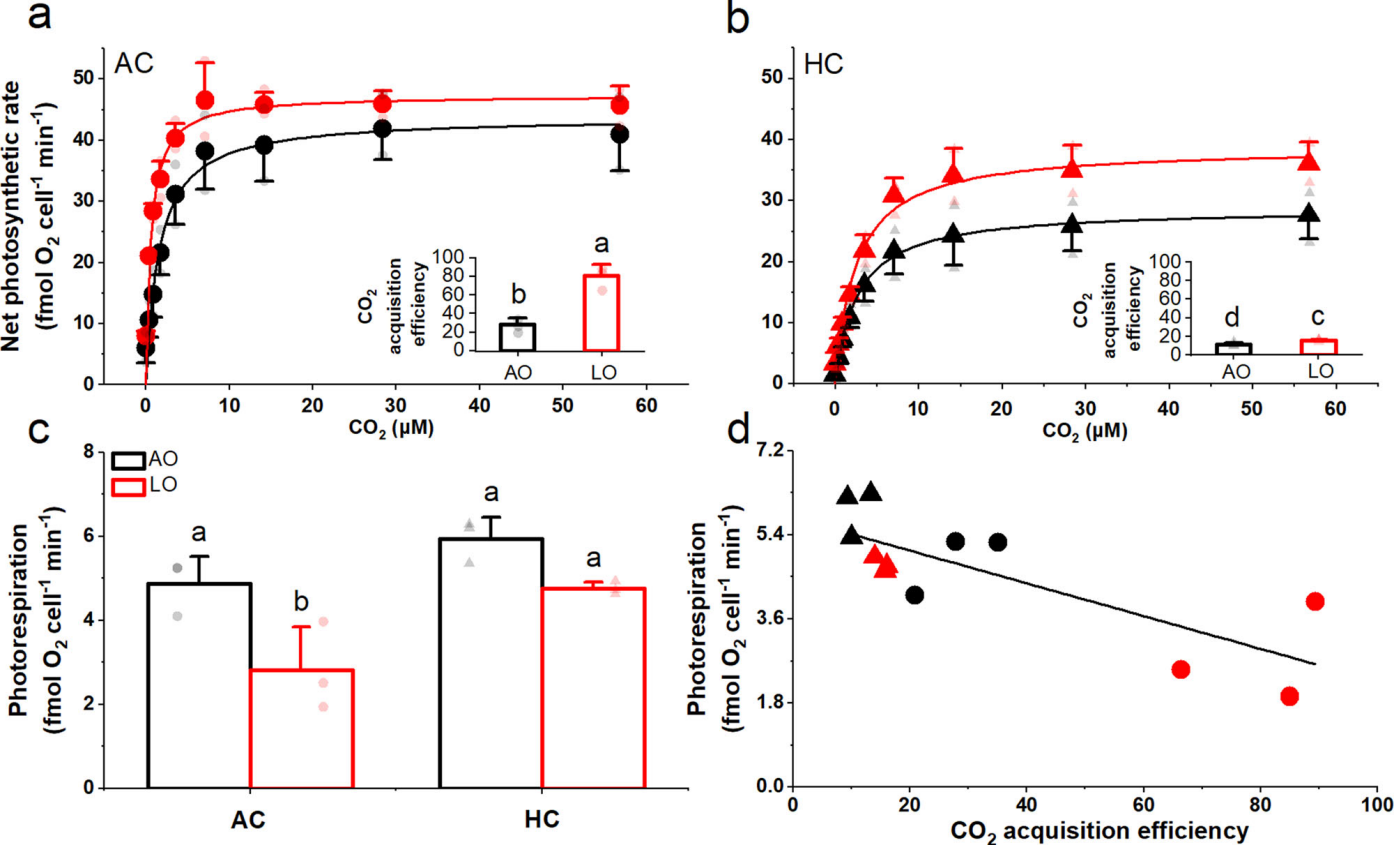
Carbon dioxide acquisition efficiencies and photorespiration rates of the diatom *Thalassiosira weissflogii* grown under varying levels of *p*CO_2_ and *p*O_2_. Net photosynthesis vs CO_2_ concentration curves was compared under standard conditions (pH_T_ = 8.00, light of 400 μmol photons m^−2^ s^−1^, and DO of 50–200 μM) for cells grown under (**a**) ambient CO_2_ (AC, circles, ~15 μM) and (**b**) high CO_2_ (HC, triangles, ~35 μM) at ambient (AO, black symbols, ~255 μM) and low O_2_ levels (LO, red symbols, ~57 μM). Insets present CO_2_ acquisition efficiencies (*V*_max_/*K*_0.5_ for CO_2_). (**c**) Photorespiration of cells under the growth *p*CO_2_ and *p*O_2_ levels. (**d**) The correlation between photorespiration and CO_2_ acquisition efficiency (*P* = 0.0023, *r* = −0.7886, Pearson correlation analysis, two-tailed). The values represent means (**a**–**c**) or all replicate data (**d**), and error bars indicate the standard deviations of independent biological replicates (*n*  =  3). Light-colored symbols are individual data corresponding to the treatments. Different letters above the bars represent significant differences (*P* < 0.05, LSD test) among the treatments. The detailed information for the experimental features and timing points for the above determinations are shown in Supplementary Fig. 3.

To explore the mechanisms involved, we tested the CCM capacity of the diatom cells acclimated to different combinations of *p*O_2_ and *p*CO_2_ using direct comparisons under standard conditions (pH_T_ = 8.00, 50–200 μM O_2_). The LOAC-grown cells had a significantly lower half-saturation constant (*K*_0.5_) for CO_2_-dependent photosynthesis (Supplementary Fig. 4c and Fig. 6a, *P* < 0.0001), indicating an increased photosynthetic affinity for CO_2_ and an increase in CCM activity. Conversely, the HC-acclimated cells grown under both AO and LO levels had lower CO_2_ affinities and CCM activities, as revealed by their increased *K*_0.5_ values compared to the AOAC-grown cells (Supplementary Fig. 4c and Fig. 6a, b, *P* = 0.0038, *P* < 0.0001). The efficiency of CO_2_ acquisition, expressed here as the quotient of maximal photosynthetic rate (*V*_max_) to *K*_0.5_, increased significantly with decreased O_2_ by up to 187%, under the AC level (Fig. 6a, inset, *P* = 0.0001), but only by about 40% under the HC level (Fig. 6b, inset, *P* = 0.0481). This implies opposing effects of reduced O_2_ and elevated CO_2_ (lowered pH) on CO_2_ acquisition efficiency.

It appeared that the LO-acclimated cells increased their photorespiration when measured under the standard conditions (nearly ambient O_2_ level) that at least partly repressed net photosynthetic O_2_ evolution (Figs. 5a and  6a, b). To check if the divergences between conditions for physiological tests and for experimental cultures may potentially make the observed CCM-related photosynthetic traits under the standard conditions inaccurately reflect those under growth conditions, we examined the activity of periplasmic carbonic anhydrase (eCA) involved in the extracellular conversion of bicarbonate to CO_2_ using acetazolamide (AZ, as an inhibitor of eCA)_._ The inhibition of photosynthetic O_2_ evolution by AZ measured under culture conditions was taken as a proxy of eCA-functional capacity and CCM activity (a greater inhibition of eCA relates to higher involvement of biophysical CCMs in photosynthesis). Inhibition was significantly greater in the LO-grown cells compared to AO-grown ones (Table 1, *P* = 0.0239). This indirectly supports the results showing that lowered O_2_ concentration enhanced activity of the CCMs and CO_2_ acquisition efficiency (Supplementary Fig. 4c and Fig. 6a, b). In addition, under the HC conditions AZ had an insignificant effect on the cells grown under both O_2_ levels (Table 1), as revealed by unchanged net photosynthetic rates under both AO (*P* = 0.4982) and LO (*P* = 0.3838) conditions with AZ compared with that without AZ. This reflects that the elevated CO_2_ alone was sufficient to cause downregulation of eCA and activity of CCMs, regardless of O_2_ levels, leading to undetectable AZ impacts.

**Table 1:** Net photosynthetic rates of the diatom *Thalassiosira weissflogii* grown and measured under four *p*O_2_/*p*CO_2_ conditions with and without the extracellular (periplasmic) carbonic anhydrase inhibitor acetazolamide (AZ).

		-AZ	+ AZ	Inhibition
		(fmol O_2_ cell^−1^ min^−1^)	(fmol O_2_ cell^−1^ min^−1^)	(%)
AC	AO	41 ± 2.9^b^	36 ± 2.3^cd^	11 ± 1.6^b^
	LO	45 ± 3.5^b^	37 ± 2.6^c^	18 ± 2.1^a^
HC	AO	43 ± 2.7^b^	44 ± 3.0^b^	None
	LO	55 ± 2.1^a^	56 ± 2.3^a^	None

Values represent means ± standard deviation (*n* = 3) for replicate cultures under ambient (AO, ~255 μM) and low O_2_ levels (LO, ~57 μM) with ambient (AC, ~15 μM) and elevated CO_2_ levels (HC, ~35 μM), and different letters (superscripted) represent significant differences (*P* < 0.05, LSD test) among +AZ and −AZ treatments. Photosynthetic inhibition in high CO_2_-grown cells was not detected (none), while low O_2_-grown cells showed the highest photosynthetic inhibition. The detailed information for the experimental features and timing points are shown in Supplementary Fig. 3.

Photorespiration of the diatom declined significantly (42%) in the LO-grown cells under AC levels (*P* = 0.0058), but decreased to a much lesser extent (20%) in cells grown under LO and HC levels (Fig. 6c, *P* = 0.0637). Once again, the effects of elevated CO_2_ were opposite to the positive influence of reduced O_2_. Photorespiration correlated inversely with CO_2_ acquisition efficiency (Fig. 6d, *P* = 0.0023, *r* = −0.7886), implying a shift from oxygenation to carboxylation catalyzed by ribulose-1,5-bisphosphate due to low O_2_ enhanced CCMs activity.

On the other hand, reduced O_2_ availability under AC slightly increased the production rates of particulate organic carbon (POC), particulate organic nitrogen (PON), and biogenic silica (BSi) by 5%, 9%, and 9%, respectively (Supplementary Table 7, *P* = 0.3247, 0.1102, 0.0057). In comparison, under HC conditions, a reduced O_2_ level increased POC, PON, and BSi production respectively by 12%, 13%, and 13% (*P* = 0.0453, 0.3586, 0.0024, respectively). The C:N ratios of the cells were not altered by the LO treatments regardless of the CO_2_ levels (*P* = 0.5345, 0.9254).

## Discussion

**Fig. 7:**
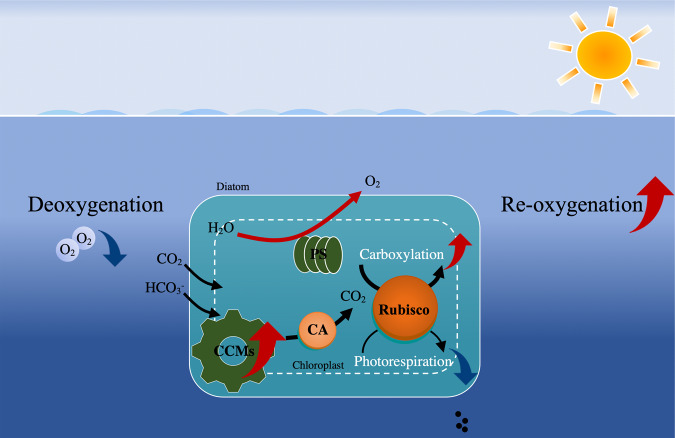
Simplified illustration of low O_2_-enhanced CCMs activity and subsequently increased re-oxygenation due to global deoxygenation and/or in intruded hypoxic waters. Reduced O_2_ levels enhance CO_2_ concentrating mechanisms (CCMs) and photosynthetic performance with increased light energy use efficiency in diatoms and phytoplankton assemblages (Figs. 2–6), promoting photosynthetic carbon fixation and O_2_ evolution. The enhanced photosynthesis and reduced mitochondrial and photorespiration (Figs. 5 and 6) can accelerate the re-oxygenation due to stimulated photosynthetic O_2_ evolution by up to 193–250% (based on net photosynthetic values of day 5 in Fig. 2 assuming that the photosynthetic quotient is 1.0). In natural environments, low O_2_-enhanced production of phytoplankton biomass makes them a more effective O_2_ source that may help to counteract the negative effects of hypoxia on heterotrophs. Black, red, and blue arrows indicate directions, increase and decrease, respectively.

We found that reduced levels of dissolved O_2_ increased primary productivity of natural phytoplankton assemblages and stimulated growth and enhanced photosynthetic performance with increased activity of CCMs in a cultured diatom (Fig. 7). Mechanistically, low O_2_-enhancement of CCMs activity along with improved light use efficiency and the reduction in photorespiration allow low O_2_-grown phytoplankton to perform more efficient photosynthetic carbon fixation (Figs. 2 and 5) and result in faster growth in the diatom (Fig. 5). Reduced photorespiration from favored carboxylation may increase the demand for inorganic carbon, and the reduced mitochondrial respiration may result in decreased intracellular CO_2_ supply through the respiratory pathway and thus enhance the CCM activity of cells grown under low O_2_ levels. Although the antagonistic effects of increased CO_2_ projected for the end of this century on CCMs partly canceled out the positive effects of decreased O_2_ on the diatom (Fig. 6 and Table 1), reduced levels of O_2_ still significantly promoted their growth even under the elevated CO_2_ conditions. Suppression of respiratory carbon loss (Fig. 5) might have also contributed to the enhanced growth rates of the low-O_2_ grown diatom due to suppressed mitochondrial respiration, the rate of which depends on O_2_ levels^13^. Especially under high CO_2_ conditions, low-O_2_ grown diatoms possessed higher growth rates with lower mitochondrial respiration, implying that the energy saved from the down-regulated CCMs could have supported the energetic demands for growth so that mitochondrial respiration diminished. These findings supported our hypothesis.

Whether the positive effects of reduced O_2_ on phytoplankton assemblages observed in this work are true for dynamic in situ environments remains to be explored, in view of possible synergistic or antagonistic effects of multiple drivers. The sensitivity of phytoplankton to O_2_ can be closely linked to their physiological conditions, types and/or efficiencies of CCMs and Rubisco^19,33,34^. Thus additional environmental stresses and diverse phytoplankton assemblage structures may complicate overall ecosystem responses^35^. For instance, changes in nutrient availabilities and phytoplankton communities in our mesocosms under fluctuating levels of PAR and temperature appeared to have affected the interactions of CO_2_ and O_2_ (Figs. 2–4). In addition, other components of the plankton communities, such as grazers, might have complicated the interactions within the mesocosm system. These factors may be at least partially responsible for observed differences in the magnitude of low-O_2_ enhancement effects and high CO_2_ dampening impacts on photosynthetic carbon fixation.

Most dinoflagellates are characterized by only moderately efficient CCMs and high O_2_ affinity-form II Rubisco, and therefore may benefit more from reduced O_2_^19^. This may account for the increased proportion of dinoflagellates in our low O_2_ mesocosms after nutrients, especially after SiO_3_^2−^ became exhausted. On the other hand, their complex nutritional modes, such as heterotrophic nutrition and phagotrophy, may give dinoflagellates more strategies to withstand low O_2_ environments. As recently reported, *Noctiluca scintillans*, which relies on ingested endosymbionts, bloomed during a hypoxic event in the Arabian Sea^36^. The aforementioned positive effects of lowered O_2_ and multiple nutritional modes might have increased the abundance of dinoflagellates encountering hypoxic waters. This implies that hypoxic waters or ocean deoxygenation could enhance the development of harmful dinoflagellate blooms.

As global warming and eutrophication have perturbed the O_2_ budget of the ocean, degradation of habitat fitness for aerobic marine organisms has occurred both regionally and globally^3,4,8^. Importantly, recently reported time-series data suggest the occurrence of upwelling-induced continuous hypoxia events (~1–2 weeks) in shallower layers^37^. In our study, however, natural phytoplankton assemblages and the diatom *T. weissflogii* benefited from reduced O_2_ concentrations that were low enough to be detrimental for most marine animals^15,38^. Accordingly, even under elevated CO_2_ conditions, low O_2_-enhanced photosynthesis can accelerate “re-oxygenation” in illuminated waters by ~193–250% (based on the net photosynthetic values of day 5 in Fig. 2, and an assumption that the photosynthetic quotient is 1.0), and thus may progressively alleviate the impacts of diminished oxygen on animals (Fig. 7). Considering that open ocean diatoms are more sensitive to rising CO_2_ than coastal ones^39^, the combined impacts of reduced O_2_ and increased CO_2_ levels on coastal and pelagic phytoplankton taxa are expected to differ in extent. Thus, the present result, showing that lowered availability of O_2_ enhanced primary production of phytoplankton, indicates a possible negative feedback effect on ocean deoxygenation.

Marine primary producers are exposed to multiple stressors along with progressive ocean acidification (OA) and warming^40^, being affected by enhanced nutrient limitation in pelagic waters and by deteriorating eutrophication in coastal areas, along with ocean-warming-induced decrease in oxygen solubility. Ocean deoxygenation has been predicted to cause a further 1–7% decline in the global ocean O_2_ inventory over this century, due to global warming^8^. Moreover, increasing discharges of nitrogen and phosphorus to coastal waters^41^ and strengthening upwelling-favorable winds^42^ may make invasions of hypoxic waters into the euphotic zone happen more frequently. This has been suggested to intensify the combination of DeO_2_ and OA effects, especially in coastal regions^4,43,44^. With progressive ocean climate changes, DeO_2_ is believed to disrupt the balance between O_2_ availability and metabolic O_2_ demand of some marine biota and impact heterotrophic processes^3^. Thus, climate change (such as warming) may increase the energy demand of aerobic organisms while DeO_2_ reduces the O_2_ supply. However, both DeO_2_ and OA occur in concert with other environmental drivers and biological factors. Therefore, it is important to note that the results from our laboratory and mesocosm experiments can only provide a mechanistic understanding of the positive effects of lowered O_2_ under influence of elevated CO_2_ (Fig. 7). Other key biological responses under multiple drivers along with long-term selection and evolution of dominant phytoplankton to life under low O_2_/high CO_2_ conditions are unknown, but should be a priority for further research. Future studies on the ocean deoxygenation effects are also encouraged to include more drivers, to better reflect the real complexities of future ocean environments.

## Methods

### Field studies

Photosynthetic carbon fixation was investigated at eight different stations in the Pearl River estuary of the South China Sea (Fig. 1a and Supplementary Table 1), where the phytoplankton assemblages were dominated by diatoms^45^ during the time of our investigation (June 2015). Samples were collected from 10 to 20 m depths and transferred immediately into 50 mL quartz tubes and sealed to prevent gas exchange. The samples were inoculated with 100 μL of 5 μCi (0.185 MBq) NaH^14^CO_3_ solution for 2.15 h. All the incubations were carried out under incident solar radiation, attenuated with neutral density filters to simulate light intensities at the sampling depths, and the temperature was controlled with flow-through surface seawater.

After incubation, the cells were filtered onto glass-fiber filters (25 mm, Whatman GF/F, USA) and stored at −20 ° C until measurement, during which the filters were exposed to HCl fumes overnight and dried (20 °C, 6 h) to remove unincorporated NaH^14^CO_3_ as CO_2_. The incorporated radioactivity was measured by liquid scintillation counting (LS 6500, Beckman Coulter, USA), and photosynthetic carbon fixation rates were estimated as previously reported^46^. Since the measurements were carried out under varying and low light levels similar to in situ levels at depths of 10 and 20 m, we normalized the photosynthetic rates to light intensity (μmol C (μg Chl *a*)^−1^ h^−1^ (μmol photons m^−2^ s^−1^)^−1^) to obtain the light use efficiency of photosynthesis (PLUE). This was done to allow for a meaningful comparison among different stations according to the linear relationship of photosynthetic carbon fixation under low solar irradiance levels^46^, which lies within the range of sunlight levels used in the present fieldwork (<100 μmol photons m^−2^ s^−1^).

Field DO, chlorophyll *a* (Chl *a*) concentration and nutrients were measured as described previously^5,47^. Briefly, field DO was manually measured on board using the Winkler titration method^48^. The Chl *a* content was measured with a Turner Designs Model 10 Fluorometer. The nitrogen (NO_X_, NO_3_^−^ + NO_2_^−^), NH_4_^+^, and SiO_3_^2−^ concentrations were measured with a nutrient-autoanalyzer (Quickchem 8500, Lachat Instruments, USA) following the description of Kirkwood et al.^49^. This equipment has detection limits of 0.014 and 0.075 μM for NO_X_ and SiO_3_^2−^, respectively.

Dissolved inorganic carbon (DIC) concentrations at investigated stations were estimated based on measured salinity and the relationship between salinity and DIC concentrations in the published literature^50^ in the same area of the Pearl River estuary during the same season. CO_2_ concentration and pH_T_ were calculated using CO_2_SYS software^51^, using the equilibrium constants K_1_ and K_2_ for carbonic acid dissociation^52^.

### Mesocosm studies

Surface seawater (0–1 m) with natural plankton assemblages was sampled from a harbor near the Dongshan Swire Marine Station of Xiamen University (23.65^o^ N, 117.49^o^ E) with an acid-cleaned plastic bucket, filtered (180 μm) to remove large grazers, and transported to the station within 1 h. The incubation system used 30-liter cylindrical polymethyl methacrylate tanks (*n* = 3), which allowed 91% PAR transmission and were water-jacketed for temperature control with a re-circulating cooler (running water). We set two O_2_ and two CO_2_ levels with three *p*O_2_:*p*CO_2_ combinations: (1) ambient O_2_ (AO, ~213 μM) & ambient CO_2_ (AC, ~13 μM), AOAC; (2) low O_2_ (LO, ~57 μM) & ambient CO_2_, LOAC; (3) low O_2_ & high CO_2_ (HC, ~27 μM), LOHC. The presented O_2_ and CO_2_ concentrations are average values across the entire experiment. N_2_, CO_2_, and air were mixed proportionally to create different and stable *p*O_2_:*p*CO_2_ combinations in the gas stream. The incubation tanks were continuously aerated (0.5 L min^−1^) under incident solar radiation. The O_2_ concentration was measured (20:00) with a precise single-channel fiber optic oxygen sensor (Microx 4, PreSence, Germany) every day. CO_2_ concentrations of seawater were calculated from daily measured pH_NBS_ (20:00) and TA measured every other day using CO_2_SYS software. The pH was determined according to Dickson (2010)^53^ with a high-quality pH meter (Orion StarA211, Thermo, USA) which was calibrated with standard National Bureau of Standards (NBS) buffer solutions (Hanna). The pH_NBS_ values were converted to pH_Total_ (pH_T_) using the CO_2_SYS software as described above.

For nutrient measurements, water samples were stored in 80-mL polycarbonate bottles, instantly frozen, and stored at −20 °C until analysis. Samples for silicate determination were fixed with 1‰ chloroform and preserved at 4 °C. Nutrients were measured with an AA3 Auto-Analyzer (Bran-Luebbe, GmbH, Germany) with detection limits of 0.08, 0.08, and 0.16 μM for NO_X_, PO_4_^3−^, and SiO_3_^2−^, respectively.

Samples for analysis of Chl *a* and other pigments were filtered onto glass-fiber filters (25 mm, Whatman GF/F, USA) which were immediately preserved in liquid nitrogen until analysis. Measurement was conducted with a high-performance liquid chromatography system (UltiMate 3000, ThermoFisher Scientific, USA) after filters were submerged in N, N-dimethylformamide and then mixed 1:1 (V:V) with 1-M ammonium acetate^54^. Chlorophyll *a* and other pigments were identified by their retention times and quantified using peak areas and standard curves. Quantification was performed with standards purchased from DHI Water & Environment, Hørsholm, Denmark. Chemotaxonomic analysis was carried out using CHEMTAX software^55,56^.

To measure gross and net primary productivity, respectively, seawater samples were inoculated with 200 μL of 10 μCi (0.37 MBq) NaH^14^CO_3_ solution (ICN Radiochemicals, USA) for 2 h (gross) and with 100 μL of 5 μCi (0.185 MBq) NaH^14^CO_3_ solution for 24 h (net). All the incubations were carried out under incident solar radiation in a flow-through water bath to obtain a uniform temperature. Photosynthetic carbon fixation rates in the mesocosm experiment were estimated as described above.

Photosynthetic fluorescence parameters were measured with a fluorescence induction and relaxation system (In-Situ FIRe, Satlantic, NS Canada). NPQ was estimated by the equation of Genty et al.^57^:

1 $${{{{{\rm{NPQ}}}}}}=({{{{{{\rm{F}}}}}}}_{{{{{{\rm{md}}}}}}}{-{{{{{\rm{F}}}}}}}_{{{{{{\rm{m}}}}}}}\hbox{'}){/{{{{{\rm{F}}}}}}}_{{{{{{\rm{m}}}}}}}\hbox{'}\ ,$$

where F_md_ is the maximal fluorescence measured before sunrise and F_m_’ is the effective yield at 11:00 a.m. under incident sunlight.

### Diatom culture studies

The diatom *Thalassiosira weissflogii* (CCMP 1336) was incubated in artificial seawater prepared according to the Aquil* medium recipe^58^, and was cultured semi-continuously in polycarbonate bottles. Cultures were incubated at 20 °C in a plant growth chamber (HZ100LG, Ruihua, Wuhan, China) and illuminated with cool white fluorescent light at 200 μmol photons m^−2^ s^−1^ (measured by a US-SQS/WB spherical micro quantum sensor; Walz, Germany) with a 12:12 h light:dark cycle. The maximum cell concentration was maintained below 5000 cells mL^-1^ by diluting the cultures every 24 h with newly prepared medium, equilibrated with the target O_2_ and CO_2_ levels, in order to maintain a stable range of dissolved O_2_ (DO) and carbonate chemistry in the culture without aeration (Supplementary Fig. 3). To avoid the cells settling, the bottles were shaken gently every 2 h during the daytime (0800–2000).

The diatom cells were acclimated to four treatments with two levels of CO_2_ (ambient and high CO_2_) and two levels of O_2_ (ambient and low O_2_), respectively. In order to create the ambient O_2_ & ambient CO_2_ seawater (AOAC, ~254 μM O_2_, ~15 μM CO_2_) or ambient O_2_ & high CO_2_ seawater (AOHC, ~256 μM O_2_, ~33 μM CO_2_), we aerated the medium with ambient air or CO_2_-enriched air using a CO_2_ enricher (CE-100, Ruihua, Wuhan, China). In order to maintain low O_2_ conditions and to sustain constant carbonate chemistry, pure nitrogen was introduced into the headspace of bottles containing seawater with different CO_2_ concentrations, so that the O_2_ in the water was displaced, and reduced O_2_ & ambient CO_2_ (LOAC, ~58 μM O_2_, ~14 μM CO_2_) or reduced O_2_ & high CO_2_ (LOHC, ~56 μM O_2_, ~36 μM CO_2_) conditions were achieved (Supplementary Fig. 3e, f and Supplementary Table 6).

The dissolved O_2_ and pH of seawater were measured before and after diluting the culture medium (Supplementary Fig. 3 and Supplementary Table 6). The dissolved O_2_ was measured with a Clark-type oxygen electrode (Hansatech, UK). Parameters of the seawater carbonate system (Supplemental Table 6) were calculated from pH and TA with CO_2_SYS software, and the pH_NBS_ values were converted to pH_Total_ (pH_T_) using the CO_2_SYS software as described above. Photosynthesis vs CO_2_ curves (*n* = 3) and other parameters (*n* = 3) were obtained from two separate experiments under the same experimental conditions after the cells had acclimated for at least nine generations (see Supplemental Fig. 3 for detail).

Cell concentrations were measured with a Counter Particle Count and Size Analyzer (Z2, Beckman Coulter, USA) before and after the dilutions every 24 h. The cells had acclimated for at least nine generations before the growth rate was measured. The specific growth rate (μ, d^−1^) was calculated as

2 $${{{{{\rm{\mu }}}}}}=({{{{{{\rm{lnN}}}}}}}_{1}-{{{{{{\rm{lnN}}}}}}}_{0})/({t}_{1}{-t}_{0}),$$

where N_1_ and N_0_ represent cell concentrations at *t*_1_ (before the dilution) and *t*_0_ (initial or just after the dilution), respectively.

A Clark-type oxygen electrode was used to measure mitochondrial respiration (after acclimation for ~13 generations) under the conditions of pH, O_2_ levels, and temperature used for growth, and the oxygen consumption rates were monitored in the dark (~10 min). About 6–8 × 10^5^ cells were harvested by gentle vacuum filtration (<0.01 MPa) onto polycarbonate membrane filters (1.2 μm, Millipore, Germany). These cells were then re-suspended in seawater (2 mL) buffered with 20 mM Tris (without introducing additional DIC into media, pH_T_ = 8.00 for AC of 14 μM and pH_T_ = 7.70 for HC of 34 μM) to maintain stable pH in the media. Tris-buffered seawaters were flushed with pure nitrogen and ambient air to achieve the culture O_2_ levels.

During the measurements of photosynthetic O_2_ evolution and photorespiration, 5–6 × 10^5^ cells were harvested after acclimation for ~18 generations and re-suspended as above. Photosynthetic O_2_ evolution was tested under growth O_2_ levels (~255 μM for AO and ~57 μM for LO), and photorespiration (Supplementary Fig. 5) was estimated as the difference in photosynthetic O_2_ evolution of the cells under reduced (~25 μM) and culture (~255 μM for AO and ~57 μM for LO) O_2_ conditions, an approach which has been used widely^26,59^. However, this method might have overestimated the absolute value of photorespiration to some extent because of the ignored mitochondrial respiration rates at different O_2_ levels. Therefore, we re-estimated the photorespiration (Fig. 6c) using the differences of dark-respiration rates between the samples measured under ~25 μM O_2_ and growth O_2_ conditions (~255 μM O_2_ for AO and ~57 μM O_2_ for LO), assuming that the mitochondrial respiration rates for the cells grown under the treatments were the same under light and darkness. To obtain the reduced or ambient levels of O_2_, pure nitrogen gas or ambient air were bubbled into Tris-buffered seawater (20 mM, pH_T_ = 8.00 for AC of about 14 μM and pH_T_ = 7.70 for HC of about 34 μM). Light intensity and temperature were the same as in the growth experiment.

Inhibition of photosynthetic O_2_ evolution by acetazolamide (AZ)^60^, an inhibitor of periplasmic carbonic anhydrase (eCA), was determined with a Clark-type oxygen electrode under culture conditions. We added the AZ dissolved in 0.05 mM NaOH at a final concentration of 100 μM; an equal amount of 0.05 mM NaOH was added as a control treatment. The cells used for this test had been acclimated to the growth O_2_ and CO_2_ levels for about ten generations, and ~5 × 10^5^ cells were harvested and re-suspended in 2 mL seawater buffered with 20 mM Tris to maintain the CO_2_ partial pressures as mentioned above. O_2_ levels were achieved and controlled as above.

The photosynthesis vs CO_2_ curves was determined with a Clark-type oxygen electrode under standard conditions commonly used for CCM studies^19^. Approximately 4–10 × 10^5^ cells were harvested as above after acclimation for approximately nine generations and were re-suspended in DIC-free seawater (2 mL) medium buffered with 20 mM Tris (pH_T_ = 8.00). The concentrations of DIC in the seawater were then adjusted by adding sodium bicarbonate solution, and the final DIC concentration reached to 8 mM. DIC (μM) values were converted to CO_2_ (μM) with CO_2_SYS software. All the cells from different treatments were measured under the same standard conditions (pH_T_ = 8.00, light intensity = 400 μmol photons m^−2^ s^−1^, O_2_ was in the range of 50–200 μM, and the temperature was controlled at 20 ± 0.1 °C). CO_2_ acquisition efficiency was calculated as

3 $${{{{{\rm{CO}}}}}}_{2}\;{{{{{\rm{acquisition}}}}}}\;{{{{{\rm{efficiency}}}}}}={V}_{{{{{{\rm{max}}}}}}}/{K}_{0.5}({{{{{{\rm{CO}}}}}}}_{2}),$$

where *V*_max_ and *K*_0.5_ were calculated by fitting the photosynthetic O_2_ evolution rates at various CO_2_ concentrations with the Michaelis–Menten formula.

Measurements of chlorophyll fluorescence parameters were carried out with a pulse amplitude modulated (PAM) fluorometer (XE-PAM, Walz, Effelrich, Germany) after the cells had acclimated for ~12 generations. Effective photosystem II (PSII) quantum yield of photosystem (Yield) was measured with an actinic light level of 226 μmol photons m^−2^ s^−1^ (similar to that of the culture level). Nonphotochemical quenching (NPQ) was also measured at this actinic light intensity.

Approximately 5–8 × 10^5^ cells were harvested (~18 generations) for measuring elemental composition. Particulate organic carbon (POC) and particulate organic nitrogen (PON) were determined by filtering cells on the pre-combusted (450 °C for 6 h) GF/F filters (25 mm, Whatman), storing at −80 °C before measuring. Filters were treated with HCl fumes to remove inorganic carbon and dried before analysis on a CHNS elemental analysizer (vario EL cube, Elementar, Germany). Biogenic silica (BSi) was determined by the spectrophotometric method^61^, and the cells were harvested onto Polycarbonate filters (1.2 μm, Millipore, Germany). Production of POC, PON, and BSi was calculated by multiplying the cellular content by specific growth rate.

### Statistics and reproducibility

The data are expressed in raw form, or presented as means ± standard deviation (SD) with *n* = 3 (triplicate cultures or mesocosms). We used one-way ANOVA to assess significant differences among the treatments. Prior to analyses, data were checked for homoscedasticity. If required, data were Ln transformed, and then LSD test was used for post hoc investigation. If the data, even after transformation, did not meet the assumption for equal variance, Games–Howell tests were chosen for post hoc investigation. Linear fitting analysis was conducted with Pearson correlation analysis (two-tailed). Partial Correlation Analysis was employed to explore the net correlation between DO and photosynthetic light use efficiency in the Pearl River estuary investigation. Parameters including pH_T_, cultured temperature, DIN, SiO_3_^2−^, DIC, and CO_2_ were under control. A 95% confidence level was used in all analyses.

### Reporting summary

Further information on research design is available in the Nature Research Reporting Summary linked to this article.

## Supplementary information

Supplementary information

Description of Additional Supplementary Files

Supplementary Data 1

Reporting Summary

## Data Availability

The source data that underlying the main charts are provided as Supplementary Data 1.
