# Supplementary material for: Enhancement of diatom growth and phytoplankton productivity with reduced O2 availability is moderated by rising CO2
Source: Commun Biol. 2022 Jan 14;5:54. doi: 10.1038/s42003-022-03006-7 (PMC8760321; doi:10.1038/s42003-022-03006-7)
Supplement: Supplementary file 1 — Supplementary information [file 42003_2022_3006_MOESM1_ESM.pdf]

Supplementary Information for

Enhancement of diatom growth and phytoplankton productivity with reduced O_2 availability is moderated by rising CO_2

Jia-Zhen Sun[1], Tifeng Wang[1], Ruiping Huang[1], Xiangqi Yi[1], Di Zhang[1], John Beardall[1,2], David A. Hutchins[3], Xin Liu[1], Xuyang Wang[1], Zichao Deng[1], Gang Li[4], Guang Gao[1], Kunshan Gao[1,5*]

[1]State Key Laboratory of Marine Environmental Science & College of Ocean and Earth Sciences, Xiamen University, Xiamen 361005, China
[2]School of Biological Sciences, Monash University, Clayton, Victoria 3800, Australia
[3]Marine and Environmental Biology Section, Department of Biological Sciences, University of Southern California, Los Angeles, California 90089, USA
[4]Key Laboratory of Tropical Marine Bio-resources and Ecology, South China Sea Institute of Oceanology, Chinese Academy of Sciences, Guangzhou 510301, China
[5]Co-Innovation Center of Jiangsu Marine Bio-industry Technology, Jiangsu Ocean University, Lianyungang 222005, China

*e-mail: ksgao@xmu.edu.cn

I. Supplementary Figures

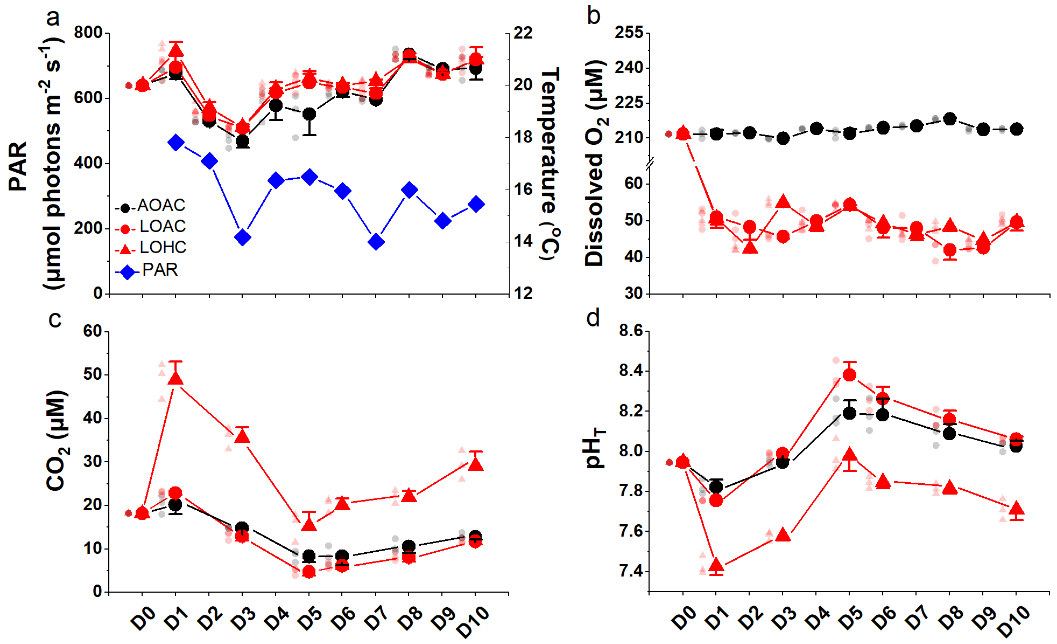

Supplementary Figure 1 (a) Daytime average PAR intensity (μmol photons m^{-2} s^{-1}, blue line) and seawater temperature, **(b)** dissolved O_2, **(c)** CO_2 and **(d)** pH_T of seawater in the 30 litre mesocosms during the experiment using coastal phytoplankton assemblages from the South China Sea during the period December 27, 2018 to January 6, 2019. D0 represents the initial time of the experiment (22:00, December 27, 2018). The values of temperature, dissolved O_2, CO_2 and pH_T values (red and black lines) are the means with error bars indicating standard deviations of independent biological replicates (n = 3 mesocosms). Light-colored symbols are individual data corresponding to the treatments. Symbols of different colors and shapes represent different treatments (**dark** indicates ambient O_2, AO; **red** indicates low O_2, LO; **circles** indicate ambient CO_2, AC; **triangles** indicate high CO_2, HC). The abbreviations for the treatments (shown in panel a) are the same as in Fig. 2.

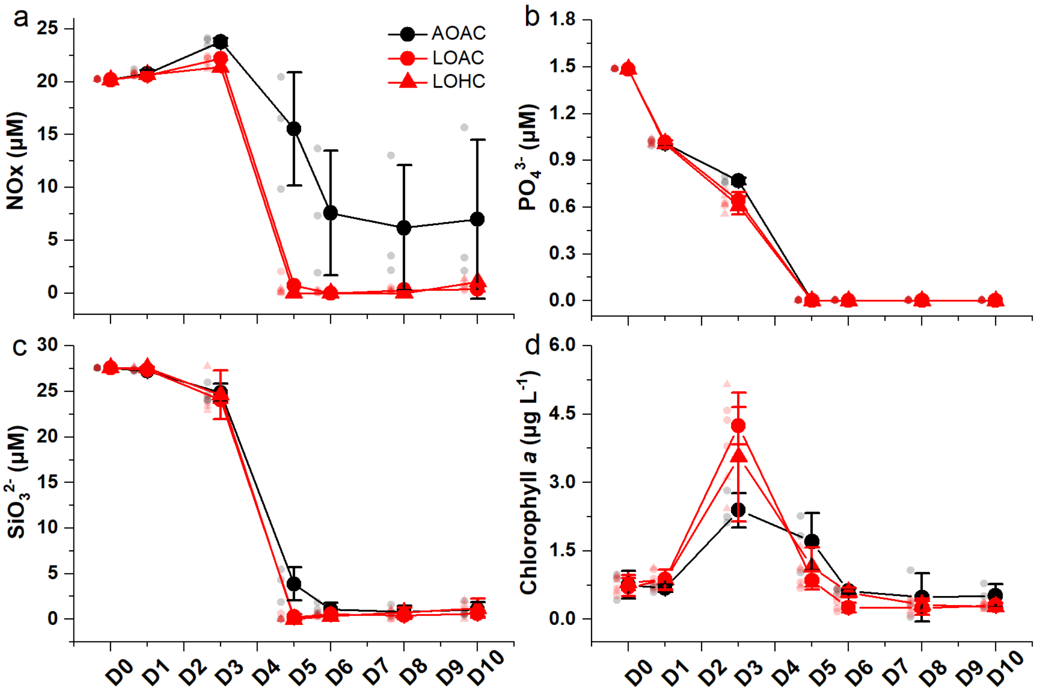

Supplementary Figure 2 Concentrations of **(a)** NO_3^- + NO_2^- (NO_X), **(b)** PO_4^{3-}, **(c)** SiO_3^{2-} (µM) and chlorophyll *a* (µg L^{-1}) in the seawater of the mesocosms under different treatments of O_2 and CO_2 (as indicated in Supplementary Fig. 1). The values are the means with error bars indicating standard deviations of independent biological replicates (n = 3 mesocosms). Light-colored symbols are individual data corresponding to the treatments. The abbreviations for the treatments (shown in panel a) are the same as in Fig. 2.

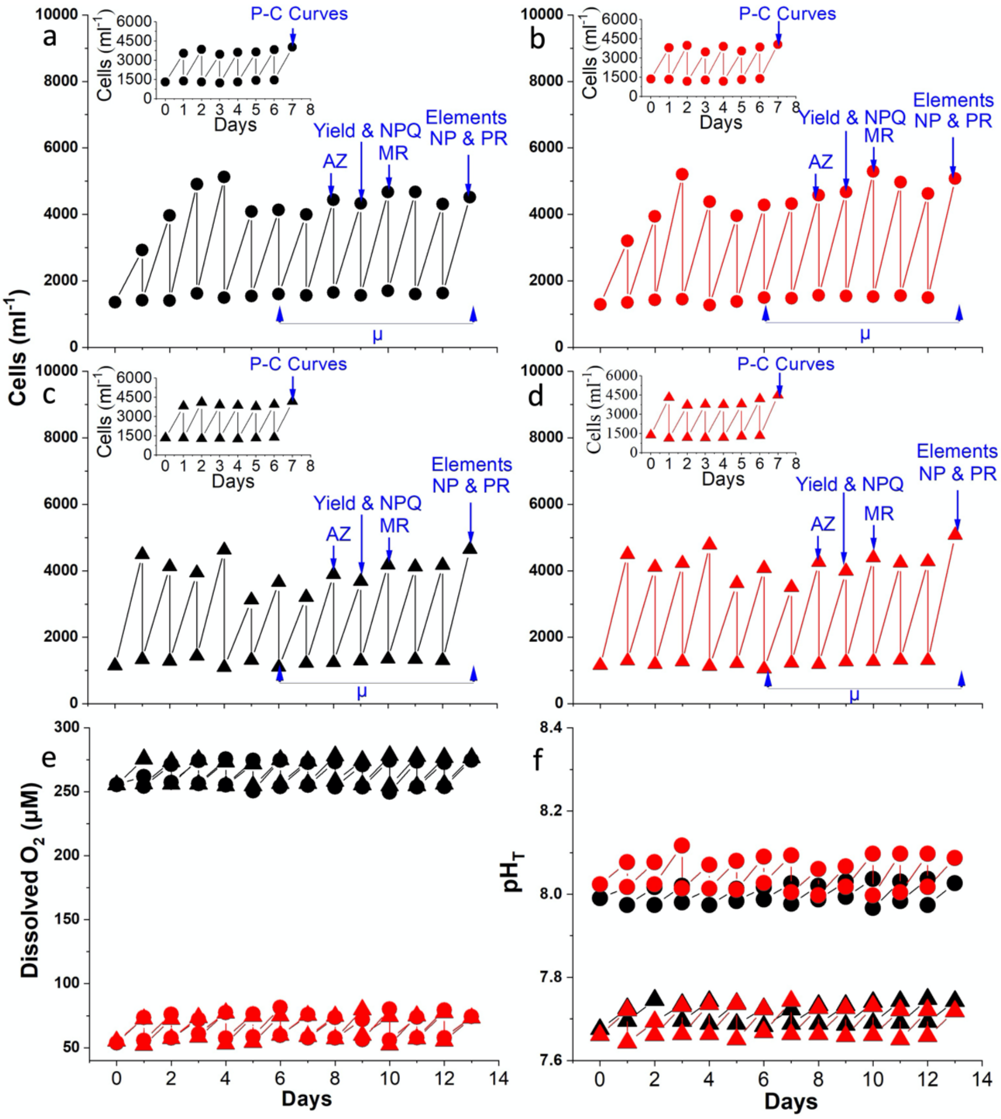

Supplementary Figure 3 Cell concentrations between the dilution intervals during the diatom *Thalassiosira weissflogii* culture experiments, and timing points at which each parameter was measured as well as the associated levels of dissolved O_2 and pH_T. Concentrations of cells that acclimated under **(a)** ambient O_2 (**black**) & ambient CO_2 (**circles**), AOAC; **(b)** low O_2 **(red)** & ambient CO_2, LOAC; **(c)** ambient O_2 & high CO_2 (**triangles**), AOHC; and **(d)** low O_2 & high CO_2, LOHC, in the diatom culture experiments. The associated **(e)** dissolved O_2 and **(f)** pH_T in the diatom-growth cultures. Note, the photosynthesis vs CO_2 curves (inserts) were obtained from a separate experiment with the same experimental conditions. Lines indicate change in cell concentrations before and after the 24 h dilution with newly prepared medium at the target CO_2-O_2 levels. P-C Curves: photosynthesis vs CO_2 curves; AZ: inhibition by AZ (acetazolamide, periplasmic carbonic anhydrase inhibitor) of photosynthesis; Yield & NPQ: photochemical yield & non-photochemical quenching; NP & PR: net photosynthetic O_2 evolution and photorespiration; Elements: particle organic carbon, nitrogen and biogenic silica; MR: mitochondrial respiration; μ: specific growth rate.

All the measurements were carried out after the diatom cells had acclimated for at least 9 generations. The values are the means of independent biological replicates (n = 3).

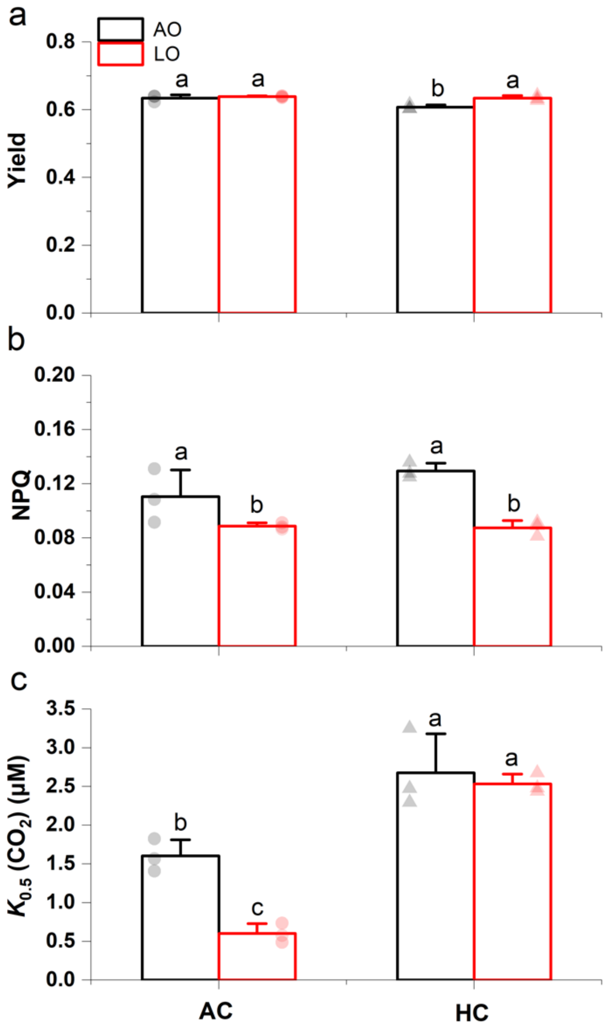

Supplementary Figure 4 (a) Effective PSII quantum yield (Yield), **(b)** non-photochemical quenching (NPQ) and **(c)** the $K_{0.5}$ values for CO_2 derived from the photosynthesis vs CO_2 curves (Fig. 6a,b) of the diatom cells grown under the combined levels of pCO_2 and pO_2 (as indicated in Supplementary Fig. 3). Yield and NPQ were measured under culture conditions with the actinic light of 226 μmol photons m^{-2} s^{-1}, which is similar to that of growth light; photosynthesis vs CO_2 curves were measured under standard condition (as indicated in Fig. 6). The values are the means with error bars indicating standard deviations of independent biological replicates (n = 3). Light-colored symbols are individual data corresponding to the treatments. Bars marked with different letters are significantly different from each other ($p < 0.05$, LSD-test).

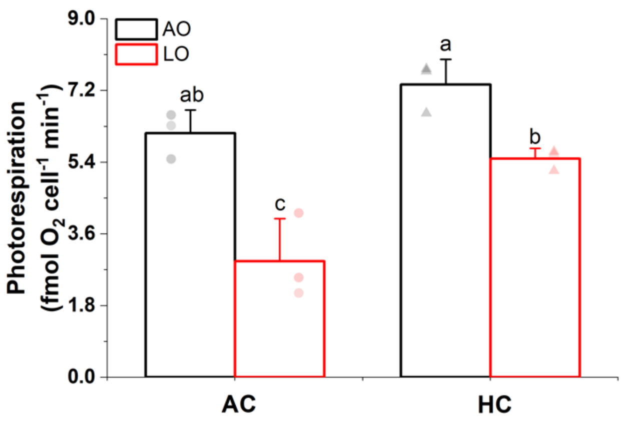

Supplementary Figure 5 Photorespiration rates that were estimated according to previous studies[26], which may ignore the difference between mitochondrial respiration under different O_2 levels when measured the net photosynthetic O_2 rates. The re-estimated values are presented in Fig. 6c. The values are the means with error bars indicating standard deviations of independent biological replicates (n = 3). Light-colored symbols are individual data corresponding to the treatments. Bars marked with different letters are significantly different from each other ($p < 0.05$, LSD-test).

II. Supplemental Tables

Supplementary Table 1 Environmental parameters for the field investigations carried out in the Pearl River estuary in June, 2015, including sampling depth (m), station numbers, salinity (‰), dissolved O_2 (DO, mg L^{-1}), pH_T, CO_2 (μM), dissolved inorganic carbon (DIC, μM), light intensity (PAR, average values during the 2.15 h incubation with C^{14}, μmol photons m^{-2} s^{-1}), field & cultured temperature (°C), DIN (NO_3^-+NO_2^- + NH_4^+, μM), SiO_3^{2-} (μM), and Chl *a* (μg L^{-1}) of phytoplankton assemblages. DIC levels were estimated using the correlation of DIC and salinity from Li et al. (2018)[50] in the same area of the Pearl River estuary during the same season.

Sampling Depth	Station	Salinity	DO	pH_T	CO_2	DIC	PAR	Cultured temperature	Field temperature	DIN	SiO_3^{2-}	Chl *a*
10 m	A3	32.44	6.30	7.91	14.22	1924.75	98.76	27.85	27.17	3.38	17.97	0.66
	B4	33.26	6.90	7.97	12.37	1928.31	86.71	28.18	29.01	5.59	34.28	0.68
	C10	33.46	5.23	7.80	18.18	1929.18	28.06	28.71	25.43	1.63	23.04	1.28
	C11	33.74	6.51	8.09	8.58	1930.40	95.29	29.89	29.45	1.74	6.65	1.02
	D4	33.45	6.55	8.09	8.53	1929.14	60.28	30.50	29.86	19.72	35.90	1.79
	E1	33.92	3.40	7.89	15.17	1931.18	25.83	27.48	23.11	27.89	23.26	2.12
	E4	33.62	6.70	8.11	8.42	1929.88	71.17	28.13	29.88	11.2	18.09	0.96
	F4	33.02	6.70	7.98	11.76	1927.27	31.35	28.41	30.40	1.80	6.25	0.49
20 m	A3	34.04	5.23	7.95	12.98	1931.70	57.22	27.85	21.98	5.53	14.71	3.10
	B4	33.53	6.56	7.97	12.34	1929.49	50.24	28.18	24.54	3.29	22.62	0.79
	C11	33.68	6.97	8.07	9.04	1930.14	55.21	29.89	26.88	3.32	29.06	0.64
	D4	33.72	7.33	8.09	8.48	1930.31	34.92	30.50	25.95	3.51	15.42	1.43
	E4	33.69	6.72	8.11	8.41	1930.18	41.24	28.13	27.87	4.39	18.24	0.70
	F4	33.39	6.52	8.02	10.59	1928.88	9.02	28.41	29.91	1.03	2.33	0.30

Supplementary Table 2 Results of Pearson correlation analysis (two-tailed) between photosynthetic light use efficiency (PLUE) and dissolved O_2 (DO), pCO_2, pH_T, dissolved inorganic nitrogen (DIN, NO_3^- + NO_2^- NH_4^+), SiO_3^{2-} in the Pearl River estuary field investigations. Partial correlation coefficients between DO and PLUE. *r* represents correlation coefficient. "*" represents significant differences ($p < 0.05$).

		Pearson correlation analysis					Partial correlation analysis
		DO	CO_2	pH_T	DIN	SiO_3^{2-}	DO
PLUE	*p*	< 0.0001	< 0.0001	< 0.0001	0.6671	0.0707	0.0035
	r	-0.6120*	0.6589*	-0.6463*	0.0684	-0.2817	-0.4736*

Supplementary Table 3 Gross photosynthesis (GP, incubated for 2 h, µmol C L^{-1} h^{-1}), net photosynthesis (NP, incubated for 24 h, µmol C L^{-1} h^{-1}) and Chl *a* (µg L^{-1}) in the 30 litre mesocosms under different treatments (abbreviations are indicated in Supplementary Fig. 1) at day 1 (D1), day 3 (D3), day 5 (D5) and day10 (D10), respectively. Values represent means ± standard deviations of independent biological replicates (n = 3 mesocosms). Gross photosynthesis was not measured (na) at day 1. Different letters (superscripted) indicate significant difference among the treatments ($p < 0.05$, LSD-test).

		D1	D3	D5	D10
GP	AOAC	na	$0.69±0.17^{a}$	$3.84±0.51^{b}$	$0.22±0.30^{a}$
	LOAC	na	$0.99±0.45^{a}$	$5.82±0.49^{a}$	$0.46±0.05^{a}$
	LOHC	na	$1.02±0.28^{a}$	$5.69±0.24^{a}$	$0.39±0.37^{a}$
NP	AOAC	$0.18±0.03^{b}$	$0.28±0.03^{b}$	$2.68±0.79^{b}$	$0.09±0.11^{a}$
	LOAC	$0.21±0.04^{ab}$	$0.48±0.10^{a}$	$4.74±0.30^{a}$	$0.21±0.00^{a}$
	LOHC	$0.23±0.01^{a}$	$0.50±0.06^{a}$	$5.33±0.91^{a}$	$0.17±0.14^{a}$
Chl *a*	AOAC	$0.68±0.08^{a}$	$2.39±0.38^{b}$	$1.70±0.63^{a}$	$0.51±0.26^{a}$
	LOAC	$0.87±0.22^{a}$	$4.24±0.41^{a}$	$0.86±0.21^{a}$	$0.30±0.05^{a}$
	LOHC	$0.87±0.22^{a}$	$3.56±1.41^{ab}$	$1.15±0.43^{a}$	$0.28±0.08^{a}$

Supplementary Table 4. Statistically significant levels (*p* values) for comparisons of net (NP) and gross (GP, in parentheses) photosynthesis among different treatments (abbreviations are indicated in Supplementary Fig. 1) at different days. Parameters that were not measured at specific time were presented as 'na'. The values from left to right columns, either based on per volume of seawater or per Chl *a*, represent comparisons for LOAC vs AOAC, LOHC vs AOAC and LOAC vs LOHC, respectively. "*" represents significant difference ($p < 0.05$, LSD-test).

		Per Volume (μmol C L^{-1} h^{-1})			Per Chl *a* (μmol C (μg Chl *a*)$^{-1}$ h^{-1})		
Day 1	NP<br>(GP)	0.1354<br>(na)	0.0416*<br>(na)	0.4233<br>(na)	0.9099<br>(na)	0.7030<br>(na)	0.6230<br>(na)
Day 3	NP<br>(GP)	0.0102*<br>(0.3094)	0.0076*<br>(0.2691)	0.8073<br>(0.9180)	0.8550<br>(0.3398)	0.1650<br>(0.8618)	0.1270<br>(0.2688)
Day 5	NP<br>(GP)	0.0124*<br>(0.0014*)	0.0040*<br>(0.0019*)	0.3534<br>(0.7411)	0.0145*<br>(0.0203*)	0.0359*<br>(0.0828)	0.5060<br>(0.3330)
Day 10	NP<br>(GP)	0.0963<br>(0.3282)	0.2416<br>(0.4723)	0.5270<br>(0.7762)	0.0529<br>(0.1656)	0.1076<br>(0.2332)	0.6246<br>(0.8092)

Supplementary Table 5 Statistically significant levels (*p* values) for comparisons of diurnal changes of photosystem II (PSII) quantum yield (Yield) and the effective functional absorption cross-section of PSII (σ_{PSII}', in parentheses) among different treatments (abbreviations are indicated in Supplementary Fig. 1) at different days. Parameters that were not measured at specific time were presented as 'na'. The values above and below the dot lines represent comparisons for LOAC vs AOAC and LOHC vs AOAC, respectively. "*" represents significant difference ($p < 0.05$, LSD-test or Games-Howell test).

		06:00	08:00	11:00	12:00	13:00	14:00	15:00	16:00	17:00	18:00
Day 4	Yield	0.0813	0.0483*	0.0075*	0.0677	na	0.0044*	0.1377	0.0664	na	0.0011*
	(σ_{PSII}')	(0.0223*)	(0.3236)	(0.0278*)	(0.2962)	(na)	(0.0042*)	(0.0080*)	(0.7898)	(na)	(0.0119*)
	Yield	0.0318*	0.0401*	0.0284*	0.0456*	na	0.0028*	0.0016*	0.0002*	na	0.0008*
	(σ_{PSII}')	(0.0557)	(0.3847)	(0.0780)	(0.0398*)	(na)	(0.0004*)	(0.0024*)	(0.0334*)	(na)	(0.0362*)
Day 8	Yield	0.6596	0.9949	0.2306	na	na	na	na	0.5136	na	na
	(σ_{PSII}')	(0.0337*)	(0.0565)	(0.2165)	(na)	(na)	(na)	(na)	(0.3215)	(na)	(na)
	Yield	0.8426	0.5759	0.7071	na	na	na	na	0.7565	na	na
	(σ_{PSII}')	(0.0053*)	(0.7796)	(0.8027)	(na)	(na)	(na)	(na)	(0.2541)	(na)	(na)
Day 10	Yield	0.6498	0.2770	0.2887	na	0.5059	na	0.0134*	0.0571	0.1706	na
	(σ_{PSII}')	(0.2144)	(0.0374*)	(0.0548)	(na)	(0.2181)	(na)	(0.0451*)	(0.0160*)	(0.0063*)	(na)
	Yield	0.9531	0.3292	0.2572	na	0.2817	na	0.5292	0.1780	0.1123	na
	(σ_{PSII}')	(0.5251)	(0.0828)	(0.1447)	(na)	(0.2309)	(na)	(0.1738)	(0.1565)	(0.0876)	(na)

Supplementary Table 6 Oxygen conditions and parameters of the seawater carbonate system for the four treatments in the diatom culture experiment. The seawater O_2 and pH were monitored every day to ensure all treatments were maintained under relatively stable culture conditions (shown in Supplementary Fig. 3). Seawater carbonate system was calculated by CO_2SYS software with pH and TA. Values represent means ± standard deviations of independent biological replicates (n = 3 cultures) during the whole experiment (13 days) except for the TA data, which were obtained independently (n = 3) and were assumed to represent conditions of the diatom culture experiment because of the same experimental treatments. S and E reflect the timing of measurements, after (S) and before (E) diluting the medium in the semi-continuous cultures, respectively. Different superscripted letters indicate significant differences (p < 0.05, Games-Howell test) between different treatments.

		pH_T	O_2 (μM)	CO_2 (μM)	HCO_3^- (μM)	CO_3^{2-} (μM)	DIC (μM)	Total alkalinity (μM)
AC	AO-S	7.98 ± 0.01^{d}	254.10 ± 2.29^{d}	15.21 ± 0.45^{e}	1887.12 ± 8.79^{e}	163.37 ± 3.49^{d}	2065.68 ± 5.77^{e}	2307.1 ± 4.33^{f}
	AO-E	8.02 ± 0.01^{b}	271.67 ± 5.04^{b}	13.58 ± 0.41^{g}	1859.14 ± 9.04^{f}	177.82 ± 3.60^{b}	2050.49 ± 5.86^{f}	2315.2 ± 4.05^{f}
	LO-S	8.01 ± 0.01^{c}	58.46 ± 5.57^{f}	13.95 ± 0.53^{f}	1864.19 ± 11.66^{f}	173.87 ± 4.61^{c}	2052.00 ± 7.60^{f}	2310.5 ± 9.77^{ce}
	LO-E	8.09 ± 0.02^{a}	73.74 ± 6.49^{e}	11.36 ± 0.64^{h}	1795.31 ± 18.41^{g}	198.06 ± 7.27^{a}	2004.74 ± 11.79^{g}	2303.1 ± 3.37^{f}
HC	AO-S	7.69 ± 0.01^{g}	255.77 ± 1.46^{c}	33.46 ± 1.19^{b}	2113.31 ± 6.85^{b}	93.17 ± 2.73^{g}	2239.99 ± 5.30^{b}	2355.6 ± 2.47^{abcde}
	AO-E	7.74 ± 0.02^{e}	275.00 ± 3.89^{a}	29.24 ± 1.27^{d}	2082.83 ± 8.97^{d}	103.61 ± 3.58^{e}	2215.73 ± 6.66^{d}	2351.3 ± 3.92^{de}
	LO-S	7.66 ± 0.01^{h}	55.93 ± 3.60^{f}	36.05 ± 1.15^{a}	2123.75 ± 5.79^{a}	87.29 ± 2.32^{h}	2247.07 ± 4.63^{a}	2351.3 ± 1.24^{c}
	LO-E	7.72 ± 0.02^{f}	72.22 ± 9.22^{e}	30.46 ± 1.33^{c}	2097.40 ± 8.52^{c}	100.91 ± 3.41^{f}	2228.76 ± 6.44^{c}	2358.8 ± 1.93^{ade}

Supplementary Table 7 Cellular quotas of particulate organic carbon (POC), particulate organic nitrogen (PON), biogenic silica (BSi), and elemental ratios and production of *T. weissflogi*i cells grown at ambient (AO, ~255 µM) and low O_2 levels (LO, ~57 µM) with ambient (AC, ~15 µM) and elevated CO_2 levels (HC, ~35 µM) for ~18 generations. Values represent means ± standard deviations of independent biological replicates (n = 3 cultures). LSD or Games-Howell tests were conducted for post hoc investigation, and different superscripted letters indicate significant differences ($p < 0.05$).

		C/N (pmol:pmol)	POC	PON (pmol cell^{-1})	BSi	POC production	PON production (pmol cell^{-1} d^{-1})	BSi production
AC	AO	9.035±0.804^{a}	20.185±0.841^{a}	2.242±0.137^{a}	1.806±0.104^{a}	20.283±1.318^{ab}	2.249±0.089^{ab}	1.812±0.070^{b}
	LO	8.649±0.539 ^{a}	18.521±0.988^{b}	2.143±0.873^{a}	1.730±0.058^{a}	21.231±1.109^{a}	2.456±0.068^{a}	1.984±0.074^{a}
HC	AO	9.512±0.357^{a}	15.965±0.675^{c}	1.679±0.025^{b}	1.316±0.018^{b}	18.236±0.582^{b}	1.977±0.184^{b}	1.504±0.010^{d}
	LO	9.453±1.030^{a}	16.373±0.909^{c}	1.741±0.152^{ab}	1.370±0.030^{b}	20.375±1.259^{a}	2.167±0.197^{ab}	1.704±0.046^{c}
