# Supplementary material for: Enhancement of diatom growth and phytoplankton productivity with reduced O2 availability is moderated by rising CO2
Source: Commun Biol. 2022 Jan 14;5:54. doi: 10.1038/s42003-022-03006-7 (PMC8760321; doi:10.1038/s42003-022-03006-7)
Supplement: Supplementary file 2 — Description of Additional Supplementary Files [file 42003_2022_3006_MOESM2_ESM.pdf]

Description of Additional Supplementary Files

File name: Supplementary Data 1

Description: The source data that underlying the main charts are provided as Supplementary Data. The Supplementary Data (Excel format) includes 6 Sheets, which are named by corresponding figures or table.
