# Supplementary material for: Enhancement of diatom growth and phytoplankton productivity with reduced O2 availability is moderated by rising CO2
Source: Commun Biol. 2022 Jan 14;5:54. doi: 10.1038/s42003-022-03006-7 (PMC8760321; doi:10.1038/s42003-022-03006-7)
Supplement: Supplementary file 4 — Reporting Summary [file 42003_2022_3006_MOESM4_ESM.pdf]

nature portfolio

Corresponding author(s): Kunshan Gao

Last updated by author(s): Nov 30, 2021

Reporting Summary

Nature Portfolio wishes to improve the reproducibility of the work that we publish. This form provides structure for consistency and transparency in reporting. For further information on Nature Portfolio policies, see our Editorial Policies and the Editorial Policy Checklist.

Statistics

For all statistical analyses, confirm that the following items are present in the figure legend, table legend, main text, or Methods section.

n/a	Confirmed	
☐	☒	The exact sample size (*n*) for each experimental group/condition, given as a discrete number and unit of measurement
☐	☒	A statement on whether measurements were taken from distinct samples or whether the same sample was measured repeatedly
☐	☒	The statistical test(s) used AND whether they are one- or two-sided<br>*Only common tests should be described solely by name; describe more complex techniques in the Methods section.*
☐	☒	A description of all covariates tested
☐	☒	A description of any assumptions or corrections, such as tests of normality and adjustment for multiple comparisons
☐	☒	A full description of the statistical parameters including central tendency (e.g. means) or other basic estimates (e.g. regression coefficient) AND variation (e.g. standard deviation) or associated estimates of uncertainty (e.g. confidence intervals)
☐	☒	For null hypothesis testing, the test statistic (e.g. *F*, *t*, *r*) with confidence intervals, effect sizes, degrees of freedom and *P* value noted<br>*Give P values as exact values whenever suitable.*
☒	☐	For Bayesian analysis, information on the choice of priors and Markov chain Monte Carlo settings
☒	☐	For hierarchical and complex designs, identification of the appropriate level for tests and full reporting of outcomes
☐	☒	Estimates of effect sizes (e.g. Cohen's *d*, Pearson's *r*), indicating how they were calculated

Our web collection on statistics for biologists contains articles on many of the points above.

Software and code

Policy information about availability of computer code

Data collection	CO2SYS software (V.2.3) was used to calculate parameters of the seawater carbonate system
Data analysis	SPSS statistics (V.21)

For manuscripts utilizing custom algorithms or software that are central to the research but not yet described in published literature, software must be made available to editors and reviewers. We strongly encourage code deposition in a community repository (e.g. GitHub). See the Nature Portfolio guidelines for submitting code & software for further information.

Data

Policy information about availability of data

All manuscripts must include a data availability statement. This statement should provide the following information, where applicable:

- Accession codes, unique identifiers, or web links for publicly available datasets
- A description of any restrictions on data availability
- For clinical datasets or third party data, please ensure that the statement adheres to our policy

The source data that underlying the graphs and charts presented in the main figures are available as Supplementary Data. The datasets generated during the current study are available from the corresponding author upon request (ksgao@xmu.edu.cn).

Field-specific reporting

Please select the one below that is the best fit for your research. If you are not sure, read the appropriate sections before making your selection.

☐ Life sciences ☐ Behavioural & social sciences ☒ Ecological, evolutionary & environmental sciences

For a reference copy of the document with all sections, see nature.com/documents/nr-reporting-summary-flat.pdf

Ecological, evolutionary & environmental sciences study design

All studies must disclose on these points even when the disclosure is negative.

Study description

This study included three types of experiments (field investigation, mesocosm and diatom culture experiments).
1. The aim of the field investigation was to investigate whether photosynthetic activity correlates with levels of dissolved O2, a factor that has seldom been considered in the context of potential effects on oceanic primary productivity. We investigated the relationship between photosynthetic light use efficiency and dissolved O2 levels of natural phytoplankton assemblages.
2. In the mesocosm study, we used natural coastal phytoplankton assemblages to test community-level responses to three pO2:pCO2 combinations (ambient CO2 & ambient O2, ambient CO2 & low O2 and high CO2 & low O2).
3. In the diatom culture study, we investigated the CCM efficiency, photosynthetic performance and growth rate of a globally distributed diatom Thalassiosira weissflogii, grown under four pO2:pCO2 combinations (ambient CO2 & ambient O2, ambient CO2 & low O2, high CO2 & ambient O2 and high CO2 & low O2).

Research sample

Natural phytoplankton assemblages in the Pearl River estuary of the South China Sea were sampled in the field study, and that in the coastal water of the northern-east South China Sea were used in the mesocosm experiment. The diatom Thalassiosira weissflogii (CCMP 1336) was used in our study because this diatom is globally distributed and has been studied extensively.

Sampling strategy

The field experiment in the Pearl River estuary used samples collected at 2 depths (10 or 20 m) from 7 separate stations . In the mesocosm study, we sampled over a period of 10 days from 3 independent mesocosms for each treatment. In our diatom culture study, the cells were maintained, in at least triplicate independent replicates, at exponential growth phase, and the culture medium was renewed every 24h. When cells had acclimated for ~9 generations , we measured a range of physiological parameters. Parameters were not all measured simultaneously but on separate days after the acclimation phase was over, as detailed in Supplementary Information.

Data collection

We used C-14 technique, Counter Particle Count and Size Analyzer (Z2, Beckman Coulter, USA), Clark-type oxygen electrode (Hansatech, UK), precise single channel fiber optic O2 sensor (Microx 4, PreSence, Germany), nutrients-autoanalyzer (Quickchem 8500, Lachat Instruments, USA), AA3 Auto-Analyzer (Bran-Luebbe, GmbH, Germany), modulated fluorometer (XE-PAM, Walz, Efflrich,Germany), fluorescence induction and relaxation system (In-Situ FIRe, Satlantic, NS Canada), high-performance liquid chromatography system (UltiMate 3000, ThermoFisher Scientific, USA), pH meter (Orion StarA211, Thermo, USA) to collect our data (Data was collected by Jia-Zhen Sun et al).

Timing and spatial scale

Diatom culture study was carried out from October 2017 to February 2018, parameters were measured after the cells had acclimated for ~9 generations;
Field experiment was carried out in June 2015 during a research cruise;
Mesocosm experiment was carried out from December 2018 to January 2019.

Data exclusions

No data were excluded from the analyses.

Reproducibility

In the mesocosms and diatom culture experiments, we designed 3 replicats, and our field ship-board tests with natural phytoplankton assemblages (collected from 10 and 20m) were repeated in several stations. The mesocosm experiments were repeated with 30 l water-jacketed tanks over a week during our experiment. Two methods (Photosynthesis vs CO2 curves and inhibitor of periplasmic carbonic anhydrase, two separate experiments) were used to test the CCM capacity of the diatom cells acclimated to different combination of pO2 and pCO2.
Three experiments were successful and consistent.

Randomization

In the diatom culture experiment, all the incubation bottles were shaken gently at least 5 times a day and placed randomly to make sure identical exposures to light and temperature.
In the mesocosm experiment, surface seawater with natural phytoplankton assemblages was mixed well and then added to different culture systems at the same time until the target volume (30 l) was reached in order to reach uniform initial condition in different mesocosms.

Blinding

Blinding was not relevant to this study. There was no subjective scoring criteria or measurements used.

Did the study involve field work? ☒ Yes ☐ No

Field work, collection and transport

Field conditions

Our field study was carried out at eight different stations (two depths) off the Pearl River Estuary in the South China Sea.
1. Station A3 (sampling depth was 10 m): salinity was 32.44, dissolved O2 was 6.3 mg L-1, cultured temperature was 27.85 Celsius degree, DIN was 3.38 µM and pHT was 7.91;
2. Station B4 (sampling depth was 10 m): salinity was 33.26, dissolved O2 was 6.9 mg L-1,, cultured temperature was 28.18 Celsius

degree, DIN was 5.59 μM and pHT was 7.97;
3. Station C10 (sampling depth was 10 m): salinity was 33.46 ‰, dissolved O2 was 5.2 mg L-1, cultured temperature was 29.84 Celsius degree, DIN was 1.63 μM and pHT was 7.80;
4. Station C11 (sampling depth was 10 m): salinity was 33.74 ‰, dissolved O2 was 6.51 mg L-1, cultured temperature was 29.89 Celsius degree, DIN was 1.74 μM and pHT was 8.09;
5. Station D4 (sampling depth was 10 m): salinity was 33.45 ‰, dissolved O2 was 6.55 mg L-1, cultured temperature was 30.5 Celsius degree, DIN was 19.72 μM and pHT was 8.09;
6. Station E1 (sampling depth was 10 m): salinity was 33.92 ‰, dissolved O2 was 3.4 mg L-1, cultured temperature was 27.48 Celsius degree, DIN was 27.89 μM and pHT was 7.89;
7. Station E4 (sampling depth was 10 m): salinity was 33.62 ‰, dissolved O2 was 6.7 mg L-1, cultured temperature was 28.13 Celsius degree, DIN was 11.2 μM and pHT was 8.11;
8. Station F4 (sampling depth was 10 m): salinity was 33.02 ‰, dissolved O2 was 6.7 mg L-1, cultured temperature was 28.41 Celsius degree, DIN was 1.8 μM and pHT was 7.98;

9. Station A3 (sampling depth was 20 m): salinity was 34.04 ‰, dissolved O2 was 5.23 mg L-1, cultured temperature was 27.85 Celsius degree, DIN was 5.53 μM and pHT was 7.95;
10. Station B4 (sampling depth was 20 m): salinity was 33.53 ‰, dissolved O2 was 6.56 mg L-1, cultured temperature was 28.18 Celsius degree, DIN was 3.29 μM and pHT was 7.97;
11. Station C11 (sampling depth was 20 m): salinity was 33.68 ‰, dissolved O2 was 6.97 mg L-1, cultured temperature was 29.89 Celsius degree, DIN was 3.32 μM and pHT was 8.07;
12. Station D4 (sampling depth was 20 m): salinity was 33.72 ‰, dissolved O2 was 7.33 mg L-1, cultured temperature was 30.5 Celsius degree, DIN was 3.51 μM and pHT was 8.09;
13. Station E4 (sampling depth was 20 m): salinity was 33.69 ‰, dissolved O2 was 6.72 mg L-1, cultured temperature was 28.13 Celsius degree, DIN was 4.39 μM and pHT was 8.11;
14. Station F4 (sampling depth was 20 m): salinity was 33.39 ‰, dissolved O2 was 6.52 mg L-1, cultured temperature was 28.41 Celsius degree, DIN was 1.03 μM and pHT was 8.02.

Location

Our field study was carried out at eight different stations off the Pearl River Estuary.
Station A3:21.61557 N, 113.1494 E;
Station B4: 21.66403 N, 113.5155 E;
Station C10: 21.86352 N, 113.8224 E;
Station C11: 21.70172 N, 113.8494 E;
Station D4: 21.75018 N, 114.1617 E;
Station E1: 22.13785 N, 114.264 E;
Station E4: 21.79325 N, 114.5009 E.
Station F4: 21.83094 N, 114.8186 E.

Access & import/export

The Peal River estuary in the northern South China Sea is a public area in China. There was no import or export of samples to or from China where all samples were collected and all experiments were done.

Disturbance

In the short-term course experiment, we only used a small water samples, so this field work did no involve any perceivable disturbance to species and habitat.

Reporting for specific materials, systems and methods

We require information from authors about some types of materials, experimental systems and methods used in many studies. Here, indicate whether each material, system or method listed is relevant to your study. If you are not sure if a list item applies to your research, read the appropriate section before selecting a response.

Materials & experimental systems

n/a	Involved in the study
☒	☐ Antibodies
☒	☐ Eukaryotic cell lines
☒	☐ Palaeontology and archaeology
☒	☐ Animals and other organisms
☒	☐ Human research participants
☒	☐ Clinical data
☒	☐ Dual use research of concern

Methods

n/a	Involved in the study
☒	☐ ChIP-seq
☒	☐ Flow cytometry
☒	☐ MRI-based neuroimaging
